# Well-being in upper secondary school students: confirming a 15-factor structure and a class-allocated feasibility trial with practice guidance

**DOI:** 10.3389/fpsyg.2026.1790035

**Published:** 2026-03-19

**Authors:** L. Francesca Scalas, Maria Luisa Pedditzi, Stefania Cuccu, Daniela Fadda, Herbert W. Marsh

**Affiliations:** 1 Dipartimento di Pedagogia, Psicologia, Filosofia, Università degli Studi di Cagliari, Cagliari, Italy; 2 Institute for Positive Psychology and Education, Australian Catholic University, Sydney, NSW, Australia

**Keywords:** factorial structure, multidimensional program, upper secondary school, wellbeing, wellbeing profile

## Abstract

The last years of secondary school might be potentially stressful for students, leading to a decrease in school and global wellbeing. This research aimed to evaluate a multidimensional program to enhance students’ wellbeing characterized by the same theoretical framework for its development and measurement. In study 1, we tested the psychometric properties of the Wellbeing Profile to adapt it to Italian upper secondary school students (*N* = 475). In study 2, we described the development and implementation of a brief and feasible class-time program to enhance wellbeing in a sample of 12 final classes of upper secondary schools. Results confirmed that the Wellbeing Profile is a valid and reliable instrument to evaluate wellbeing from a multidimensional perspective in upper secondary school students. Results of a quasi-experimental, non-randomized class design study showed protective trends for our program in relation to some wellbeing dimensions, with small, maintained improvements on selected measures at 1-month follow-up, suggesting that the proposed activities might have strengthened the ability of students to keep a positive state of mind. Even though the training needs to be applied in larger and different samples with a randomized class design, our results are promising.

## 1 Introduction

Intervention development can be challenging and requires a recursive refinement process characterized by multiple stages, from basic research to real-world implementation (Onken et al., 2014). Sometimes interventions work well in a research framework but fail into real-world context for various reasons. Moreover, a balance in efficacy (internal validity) and effectiveness (external validity) is required. In particular, evaluation of the usefulness or efficacy of interventions is not easy nor straightforward (Coster, 2013), particularly for school-based interventions (Ride et al., 2025). Major issues pertain to the use of instruments limited in reliability or validity, especially because they cannot capture the dimensions involved in the intervention. We believe that good practice might be using the same theoretical framework for the development of an intervention program and the choice of the instrument to evaluate it. In particular, the interconnection between the intervention and its measurement might be critical. For this reason, here we confirmed the factorial structure of the WB-Pro, a well-validated multidimensional instrument to evaluate wellbeing, in a sample of upper secondary school students, and, moving from relevant dimensions, we developed and applied a multidimensional program to enhance wellbeing in upper secondary school students.

## 2 Wellbeing

Wellbeing is a complex and multidimensional construct, and literature lacks a complete consensus on its definition (Herd, 2022; Iasiello et al., 2024; Marsh et al., 2020). Psychology focuses on the subjective aspects of wellbeing rather than the objective ones (González-Carrasco et al., 2019; Organisation for Economic Cooperation and Development, 2015). Different psychological perspectives have studied wellbeing. The hedonic tradition conceptualizes wellbeing mainly as satisfaction with one’s life and pleasure or absence of a negative mood (Diener, 1984; Diener et al., 2018). The eudaimonic perspective describes wellbeing as being fully functional as individuals and its roots are self-actualization, engagement, and meaning (Deci and Ryan, 2008; Keyes, 2002; Ryan and Deci, 2001; Ryff, 1989; Seligman, 2011; Waterman, 1993, 2008). Another theoretical framework defines psychological wellbeing as equivalent to positive mental health (e.g., Jahoda, 1958) and includes both eudaemonic and hedonic aspects of wellbeing. This approach has been adopted by WHO (2013), and mental wellbeing was equated with mental health, referring to a positive state, and not just the absence of mental disorders (Huppert and So, 2013; Keyes, 2002). In this perspective, wellbeing is represented by flourishing in various domains of human experience (Keyes and Waterman, 2003; Seligman, 2011).

The Wellbeing Profile (WB-Pro—Marsh et al., 2020) explores psychological wellbeing through a multidimensional and integrative perspective that considers hedonic, eudaimonic, and mental wellbeing. In this instrument, 15 dimensions are used to analyze various areas of life: Emotional stability, Engagement, Competence, Meaning, Optimism, Positive emotions, Positive relationships, Resilience, Self-esteem, Vitality, Clear thinking, Self-acceptance, Autonomy, Empathy, and Prosocial behavior. The instrument demonstrates solid theoretical and psychometric foundations in the original U.S. adult sample (Marsh et al., 2020) and in the Italian context (Scalas et al., 2023a,b), but there is a lack of studies on youth samples. Thus, one aim of this research is to validate the Wellbeing Profile for the Italian upper secondary school students.

## 3 Wellbeing in upper secondary school students

The final years of secondary education are often considered a potentially stressful developmental period (Eccles et al., 1993; Benner, 2011; Seiffge-Krenke, 2000), reflecting the many changes associated with the transition to adulthood, which involves greater autonomy and responsibility (de Moor and Christiaens, 2024). Adolescents show considerable variability in how they respond to these educational and developmental transitions, depending on the presence of protective factors that can reduce stress. These include supportive social relationships (Rueger et al., 2010; Chu et al., 2010), emotion regulation (Compas et al., 2017), and perceived competence (Caprara et al., 2008). In Italy, sense of community and school-based social support play a key protective role (Vieno et al., 2007), while family and peer relationships, together with effective emotional regulation, are crucial for preventing behavioral problems and stress (Ciairano et al., 2009; Santilli et al., 2017). Despite these protective factors, research shows that in the final years of Italian secondary school, heavy study loads, exams, performance pressure, perceived high academic demands (D’Agostino et al., 2022), and elevated parental expectations are often linked to higher levels of anxiety and depressive symptoms in adolescents (Angelini et al., 2025). Students are also required to develop new strategies to manage decisions about their future (Galton et al., 2002), including transitions to tertiary education and entry into the labor market (de Moor and Christiaens, 2024). These challenges can influence self-perceptions (Marsh et al., 2006) and self-esteem, understood as an individual’s overall sense of self-worth (Harter, 1999). For these reasons, interventions to promote wellbeing at the end of secondary education might help students deal with this complex developmental period. Marsh’s multidimensional-dimension model of wellbeing (WB-Pro—Marsh et al., 2020) can guide the identification of specific psychological dimensions to target when promoting wellbeing. These dimensions include aspects of self-confidence (e.g., self-acceptance, competence), as well as relational and emotional dimensions (e.g., positive relations, positive emotions). While these dimensions have been extensively studied in adults (Marsh et al., 2020), research on adolescent and youth wellbeing in relation to these variables is still ongoing.

School is a privileged context, an observatory, through which to discern and understand the positive potential or difficulties that students encounter throughout their development, enabling early prevention and support interventions to be implemented. According to the guidelines of APA - American Psychological Association (2014), prevention must strengthen behaviors and attitudes that promote physical and psychological wellbeing and promote practices and policies aimed at promoting physical, social, and emotional wellbeing and reducing problem behaviors and risk. Preventive interventions aimed at preadolescents and adolescents must begin with an analysis of the specific objectives to be achieved, useful and effective strategies, and the tools available in a specific local community. These interventions focus on strengthening functional skills to effectively address life tasks and daily challenges of adolescence. In the context of universal prevention, these actions translate into the promotion of transversal skills or life skills that refer to specific psychological variables such as effective communication, interpersonal relationship skills, empathy, coping with emotions, coping with stress, self-awareness, and other skills related to clear thinking, autonomy, and competence, such as problem solving, decision making, critical thinking, and creative thinking. Many of these life skills directly correspond with some of the dimensions of the WB-Pro (Marsh et al., 2020), such as: positive relationships, positive emotions, or prosocial behavior. Others describe specific behaviors, such as problem solving and decision making, that require underlying psychological abilities such as autonomy and competence. Finally, other life skills focus on thinking skills that require clear thinking, such as critical thinking, creative thinking, and meaning.

WHO - World Health Organization (1997) indicates that these skills can be learned and function as protective factors against adolescent difficulties such as relationship problems, emotional distress, academic demotivation, poor engagement, and school dropout. In this sense, promoting engagement, competence, positive emotions, optimism, meaning, prosocial-behavior and self-acceptance in school is essential. Several studies indicate that school-based life skills education is associated with improvements in adolescents’ self-esteem compared with control groups (Moulier et al., 2019). Moreover, longitudinal studies examining school-based programs focused on life skills development report improvements in participants’ self-esteem and coping skills (Srikala and Kishore Kumar, 2010), in line with World Health Organization (WHO) recommendations.

Life skills can be developed at school through specific programs that focus either directly on the promotion of life skills (WHO - World Health Organization, 1997) or on structured educational interventions targeting psychological variables relevant to adolescence, with the aim of preventing school-related problems such as disengagement, emotional and relational distress, feelings of incompetence and inadequacy, and pessimism about the future.

## 4 The present research

School-based programs and instruments to evaluate their usefulness or efficacy are often disconnected, diminishing the possibility of effectively evaluating the interventions. To overcome this issue here we conducted two studies. In study 1, we tested the psychometric properties of a multidimensional instrument of psychological wellbeing, the WB-Pro (Marsh et al., 2020), to adapt it to upper secondary school students. In study 2, moving from relevant dimensions of the WB-Pro, we developed and implemented a program aimed at enhancing wellbeing and described the results of its application in students of the final year of upper secondary school. The intervention aimed to enhance key dimensions of wellbeing and to provide students with psychological resources that may protect against decline during a particularly demanding phase of secondary education.

## 5 Study 1: factorial structure of the WB-pro in a sample of upper secondary school students

Research has documented rising levels of distress among upper secondary school students, who are exposed to multiple academic and psychosocial stressors compared to the general population (Puja et al., 2024). Student wellbeing includes the physical, psychological, and social wellness of students. However, there is no agreed-upon definition of psychological wellbeing. Although wellbeing is influenced by objective conditions, it is primarily a psychological variable, and it depends on how well a person feels their life is going (Marsh et al., 2020). Therefore, psychological wellbeing is mainly a perceived psychological condition (Pedditzi and Scalas, 2024).

Current instruments for assessing psychological wellbeing in upper secondary school do not fully capture its multifaceted nature (Viejo et al., 2018), as they mainly focus either on mental health and distress in adolescence or, more generally, on students’ life satisfaction (Lam et al., 2024). Recently, three kinds of school students’ wellbeing have been considered: hedonic, eudemonic, and integrative, combining both hedonic and eudemonic (Bruk et al., 2024). In this perspective, the WB-Pro is a resource to investigate adolescents’ wellbeing in a multidimensional and integrative perspective.

### 5.1 Method

#### 5.1.1 Participants

An initial sample of 561 students of the 10th, 11th, and 12th grades of four upper secondary schools was recruited with a non-probabilistic sampling in Sardinia (Italy). The study was approved by the Ethics Committee of the University of the authors. Informed consent and parental authorizations for minors were collected through the school Principals and their delegates. The students completed, during class, a survey on wellbeing at school (via Google Forms; -time required 40/50 min), including the WB-Pro and additional psychological correlates. A total of 68 students did not answer correctly to at least one of the nine control questions (e.g., “To this item you must answer 3”); they were eliminated since we could not be sure about their collaboration in answering the questions (see Supplementary Table S1). To have a homogeneous sample, we also eliminated students aged below 17 years (*n* = 5) or above 20 years (*n* = 10). The final sample comprises 475 students (Male = 241; mean-age = 18.54, s.d. = 0.84) attending lyceum (69%) and technical institutes (31%).^1^

#### 5.1.2 Instruments

The Wellbeing Profile (WB-Pro) is a multi-item and multidimensional instrument developed with English-speaking adults (Marsh et al., 2020) but validated also with Italian adults (Scalas et al., 2023a, 2023b). It has a solid theoretical grounding and includes aspects of hedonic and eudaimonic wellbeing. The instrument comprises 15 scales: Emotional stability (e.g., “I am emotionally balanced and even-tempered”), Engagement (e.g., “I am almost always engaged and interested in my daily activities”), Competence (e.g., “I am competent and capable in the activities that are important to me”), Meaning (e.g., “My life has a clear sense of purpose”), Optimism (e.g., “I feel very optimistic about my future”), Positive emotions (e.g., “I am happy most of the time”), Positive relationships (e.g., “I have close and secure relationships”), Resilience (e.g., “I quickly get over and recover from significant life difficulties”), Self-esteem (e.g., “I feel that I have a number of good qualities”), Vitality (e.g., “I feel full of energy most of the time”), Clear thinking (e.g., “I am able to think clearly”), Self-acceptance (e.g., “I am accepting of my own flaws and inadequacies”), Autonomy (e.g., “I feel free to do whatever I decide to do”), Empathy (e.g., “I feel others’ emotions), and Prosocial behavior (e.g., “I frequently offer help to others”). The instrument has good psychometric properties. The 15-factor structure was confirmed with CFA (Confirmatory Factor Analysis) and ESEM (Exploratory Structural Equation Modeling), and was invariant across gender, age, and education (Marsh et al., 2020; Scalas et al., 2023a). The instrument has the advantage of producing a profile on various dimensions of wellbeing, allowing for the development of personalized interventions (Marsh et al., 2020), but can also be used to gain a total score of wellbeing based on a bifactorial structure (Scalas et al., 2023a). Additionally, two formative scales have been derived from this instrument (Marsh et al., 2020) and can be used for screenings or when researchers lack enough time to administer the full version. In this study, we added an item for the clear-thinking factor (Italian: “Di solito penso in modo chiaro”; English: “Usually I think clearly”) to overcome some issues that emerged in the adults’ sample of the Italian validation of the WB-Pro (Scalas et al., 2023a). Thus, the scale results in 49 items instead of the original 48. In Table 1, factor loadings and reliability information for participants of study 1 is reported.

**Table 1:** Factor loadings and reliability of the WB-Pro.

Scale	Item	Factor loading	Omega
Autonomy	WB7	0.83	0.85
	WB12	0.89	
	WB23	0.70	
Clear thinking	WB29	0.74	0.79
	WB36	0.56	
	WB46	0.59	
	WB49	0.86	
Competence	WB5	0.70	0.86
	WB17	0.89	
	WB22	0.85	
Emotional stability	WB10	0.64	0.76
	WB39	0.83	
	WB44	0.67	
Empathy	WB4	0.71	0.73
	WB24	0.70	
	WB43	0.46	
	WB41	0.68	
Engagement	WB13	0.77	0.83
	WB19	0.77	
	WB35	0.81	
Meaning	WB2	0.71	0.85
	WB33	0.87	
	WB38	0.86	
Optimism	WB3	0.85	0.90
	WB11	0.85	
	WB45	0.91	
Positive emotions	WB27	0.86	0.92
	WB42	0.88	
	WB48	0.92	
Positive relations	WB1	0.43	0.79
	WB20	0.65	
	WB26	0.83	
	WB28	0.83	
Prosocial behavior	WB31	0.81	0.86
	WB37	0.89	
	WB40	0.76	
Resilience	WB6	0.84	0.90
	WB9	0.91	
	WB34	0.84	
Self-acceptance	WB14	0.61	0.81
	WB18	0.86	
	WB21	0.56	
	WB32	0.84	
Self-esteem	WB15	0.83	0.88
	WB25	0.87	
	WB47	0.83	
Vitality	WB16	0.93	0.94
	WB30	0.93	
	WB8	0.88	

The Satisfaction with Life Scale (SWLS—Diener et al., 1985; Pavot and Diener, 1993) is a well-known and reliable measure of life satisfaction that has been translated and used all over the world. It is a short measure, easy to administer, and has good psychometric properties. It comprises 5 items (e.g., “The conditions of my life are excellent”) measured with a 7-point Likert-type scale ranging from 1 = strongly disagree to 7 = strongly agree. Cronbach’s alpha for SWLS in our sample was 0.84.

The Adolescent Students’ Basic Psychological Needs at School Scale (ASBPNSS) of Tian et al. (2014) derived from the Self-Determination Theory of Deci and Ryan (2000, 2008) and specifically from the Basic Psychological Needs Theory (BPNT—Deci and Ryan, 2000; Ryan et al., 2021), that include the need for autonomy, relatedness, and competence, the satisfaction of which can promote positive behaviors and subjective wellbeing in people. Tian et al. (2014) have developed a new and specific measure of adolescent students’ psychological need satisfaction at school. The scale has been validated in the Italian context (Pedditzi and Scalas, 2024) and confirmed the factorial structure and showed good psychometric properties. Autonomy items focus on the student’s need to express themselves at school (five items, e.g., “I am free to arrange my studies and extracurricular activities at school”; *α* = 0.80). Competence items measure the knowledge of school-related skills and a sense of effectiveness (five items, e.g., “I am capable of learning new knowledge at school”; *α* = 0.76). Relatedness items examine the student’s need to establish good relationships in school (five items, e.g., “Teachers and classmates care about me at school” *α* = 0.92).

My Student Life Questionnaire by Soresi and Nota (2003) measures students’ satisfaction in different school and life domains. It is comprised of 26 items with a 5-point Likert response scale (1 = not at all satisfied, 5 = very satisfied) and seven scales. School Experience (seven items, e.g., “The activities that take place at school are useful and important”; *α* = 0.91); Satisfaction with decision-making autonomy (five items, e.g., “I can furnish my room as I wish”; *α* = 0.72); Relationships with Classmates (three items, e.g., “At school my classmates treat me really well”; *α* = 0.77); General situation (three items, e.g., “I am luckier than most of my classmates”; *α* = 0.76); Relationships with Family Members (four items, e.g., “I feel really good in my house”; *α* = 0.88); Satisfaction with the recognition received (two items, e.g., “My commitment is recognized at school”; *α* = 0.69); Perceived Support (two items, e.g., “In times of need and discouragement I know who to rely on”; *α* = 0.84).

The Perceived Self-Efficacy for Self-Regulated Learning Scale by Pastorelli and Picconi (2001) is a measure of school self-efficacy that derives from the Self-Efficacy Theory of Bandura (1990), and specifically from the Italian version of the Children’s Perceived Self-Efficacy Scale of Pastorelli et al. (2001). Specifically, we used 10 items referring (*α* = 0.84) to the perceived efficacy in regulating own motivation and learning activities (e.g., “How well can you finish homework assignments by deadlines?”).

#### 5.1.3 Analyses

We tested the 15-factor structure of the instrument with CFA and used robust Maximum Likelihood estimation. Several fit indices were used to evaluate the models (Hu and Bentler, 1999): Chi-square test of exact fit (*χ*^2^), Comparative Fit Index (CFI), Tucker–Lewis Index (TLI), and Root Mean Square Error of Approximation (RMSEA). Values higher than 0.90 for TLI and CFI indicate adequate fit, and values higher than 0.95 suggest excellent fit. Concerning RMSEA, values around 0.08 indicate adequate fit and values lower than 0.06 suggest excellent fit to the data. We used McDonald’s (1970) omega (*ω*) coefficient to evaluate reliability: ω = (*Σ*|λi|)^2^/([Σ|λi|]^2^ + Σδii) where λi is the factor loadings and δii is the error variance. Analyses were performed with Mplus and SPSS. To account for the missing values full-information-maximum-likelihood was used for the analyses. Information about skewness and Kurtosis of the items is reported in Supplementary Table S2 and Supplementary Figure S1.

### 5.2 Results

#### 5.2.1 Factorial structure

The 15-dimensional factorial structure of the WB-Pro tested with CFA showed adequate fit indices (CFI = 0.92; TLI = 0.91; RMSEA = 0.045, see also Supplementary Table S3), with factor loadings ranging from 0.43 to 0.93, and reliability omega indices ranging from 0.73 (for Empathy) to 0.94 (for Vitality). Examination of the latent correlations between the 15 factors of the WB-Pro (see Supplementary Table S3a), reveals that Empathy shows very low correlations with the other factors of the WB-Pro, except for Prosocial behavior (*r* = 0.67). This happened, partially, also with the Italian adult sample where the correlations were from small to moderate (Scalas et al., 2023a), but in the adolescents’ sample it emerged more clearly. Competence and Engagement seem to be highly related (*r* = 0.84) in Italian upper secondary school students; this might suggest that Italian students engage in activities when they feel competent (and vice versa). Also, Meaning and Optimism show a very high correlation (*r* = 0.87), which might suggest that Italian students can be positive about the future when they feel a meaning in their life and what they do (and vice versa). However, the factors remain distinct, showing different patterns of associations with correlates (see Table 2). For example, as expected from a theoretical point of view, Optimism is more strongly associated than Meaning with Life satisfaction (respectively *r* = 0.66, *r* = 0.62), and with Satisfaction for actual situation (respectively *r* = 0.38, *r* = 0.34), and Positive emotions (respectively *r* = 0.73, *r* = 0.64). Additionally, Competence is more strictly related to Satisfaction with acknowledgment received than Engagement (respectively *r* = 0.43, *r* = 0.37). Moreover, it should be noted that an additional ESEM model reduces latent correlations among the factors (see Supplementary Table S3b). Further studies should better explore this issue using additional and more diverse correlates, as well as objective outcomes (e.g., grades/attendance/homework).

**Table 2:** Correlations of WB-Pro dimensions with psychological school correlates of wellbeing.

WB-Pro dimensions	Satisfaction with life scale	School satisfaction	Satisfaction with decisional autonomy	Satisfaction with school peers relations	Satisfaction with actual situation	Satisfaction with family relations	Satisfaction with acknowledgment received	Satisfaction with perceived support	Regulatory self-efficacy	Basic needs-autonomy	Basic needs-competence	Basic needs-relations
Autonomy	0.457***	0.288***	0.423***	0.217***	0.259***	0.330***	0.285***	0.306***	0.268***	0.376***	0.312***	0.330***
Clear thinking	0.515***	0.275***	0.269***	0.253***	0.318***	0.300***	0.373***	0.283***	0.507***	0.263***	0.354***	0.278***
Competence	0.507***	0.330***	0.295***	0.280***	0.387***	0.317***	0.433***	0.276***	0.441***	0.278***	0.395***	0.317***
Emotional stability	0.452***	0.194***	0.203***	0.261***	0.298***	0.283***	0.227***	0.217***	0.253***	0.233***	0.205***	0.317***
Engagement	0.609***	0.389***	0.315***	0.283***	0.331***	0.327***	0.372***	0.302***	0.446***	0.372***	0.458***	0.350***
Positive emotions	0.714***	0.283***	0.332***	0.292***	0.368***	0.364***	0.284***	0.390***	0.303***	0.248***	0.351***	0.316***
Positive relations	0.392***	0.208***	0.360***	0.320***	0.218***	0.409**	0.241***	0.662***	0.209***	0.214***	0.282***	0.372***
Prosocial behavior	0.120**	0.092	0.210***	0.036	−0.023	0.105**	0.001	0.140***	0.133***	0.035	0.049	0.013
Meaning	0.619***	0.383***	0.293***	0.294***	0.340***	0.343***	0.334***	0.276***	0.350***	0.235***	0.436***	0.317***
Optimism	0.655***	0.387***	0.287***	0.304***	0.381***	0.360***	0.348***	0.315***	0.326***	0.292***	0.413***	0.328***
Resilience	0.490***	0.224***	0.224***	0.285***	0.389***	0.251***	0.256***	0.230***	0.236***	0.265***	0.245***	0.310***
Self- acceptance	0.545***	0.249***	0.330***	0.277***	0.244***	0.306***	0.250***	0.296***	0.273***	0.186***	0.228***	0.299***
Self-esteem	0.538***	0.202***	0.317***	0.232***	0.350***	0.266***	0.291***	0.278***	0.343***	0.184***	0.270***	0.225***
Vitality	0.579***	0.299***	0.267***	0.284***	0.315***	0.266***	0.272***	0.286***	0.272***	0.283***	0.334***	0.321***

Cells in gray refer to a-priori correlations expected from a theoretical point of view. In bold correlations from moderate to high in strength >0.40; ***p < 0.001; **p < 0.01.

#### 5.2.2 Wellbeing correlates in school

We explored the association between the WB-Profile dimensions and various correlates relevant for the school context in a subsample of 383 students. All the instruments used are validated in Italian and have adequate reliability scores; therefore, to avoid unnecessary complexity, we computed scale scores and measured correlations (see Table 2). *A priori* expected correlations (cells in gray in Table 2) were all confirmed by correlation values in the expected direction. For example, the autonomy scale of the WB-Pro was positively correlated with the Satisfaction for the Decisional Autonomy scale (*r* = 0.42) and the Basic Needs Autonomy scale of the ASBPNSS (*r* = 0.38).

In Table 3 are reported the correlations between the school correlates of wellbeing and the short 5- and 15-item formative scales of wellbeing. The pattern of results is similar across the two formative short versions of the instrument.

**Table 3:** Correlations of the Global dimension of wellbeing and the formative 5-item and 15-item short formative scales of the WB-Pro with psychological school correlates of wellbeing.

Psychological correlates	WB-Pro5	WB-Pro15
Satisfaction with life	0.558^***^	0.711^***^
School satisfaction	0.312^***^	0.379^***^
Satisfaction with decisional autonomy	0.292^***^	0.394^***^
Satisfaction with school peer relations	0.347^***^	0.326^***^
Satisfaction with actual situation	0.334^***^	0.422^***^
Satisfaction with family relations	0.348^***^	0.427^***^
Satisfaction with acknowledgment received	0.330^***^	0.390^***^
Satisfaction with perceived support	0.399^***^	0.389^***^
Regulatory self-efficacy	0.364^***^	0.442^***^
Basic needs-autonomy	0.302^***^	0.319^***^
Basic needs-competence	0.354^***^	0.422^***^
Basic needs-relations	0.411^***^	0.382^***^

***p < 0.001.

### 5.3 Discussion of study 1

The WB-Pro is a good instrument to evaluate wellbeing in students. It has the advantage of providing a profile on relevant wellbeing dimensions to tailor individual or group applied interventions, and it can also provide two short formative scales that can be used when researchers and practitioners have a short time or do not need a detailed profile. Our sample of upper secondary school students was small (*N* = 475) in comparison to the studies conducted with adults (*N* > 1,000); thus, future studies could involve larger and more diverse samples to perform additional tests (e.g., invariance over gender or age). For the Empathy scale, limited associations were found with the other dimensions of the WB-Pro. Similar results, but to a lesser extent, were already found with Italian adults. It should be interesting to understand if this result is related to cultural factors (regardless of the age of participants), since it does not seem to emerge in the American sample (Marsh et al., 2020). At least in the Italian sample, the items seem to imply emotional entanglement (“Other people’s misfortunes usually disturb me a great deal” “Le disgrazie degli altri di solito mi turbano molto”) and are polarized on negative emotions (“My heart goes out to people who are unhappy” “Mi sento vicino alle persone infelici”), thus people who have high scores on this scale might be very sensitive to negative experiences of others and thus find themself in a condition of low wellbeing as a result.

Other specific results that emerged for our students’ sample are the strong associations between Competence/Engagement and Meaning/Optimism. These scales are generally correlated but to a lesser extent in adults (Marsh et al., 2020; Scalas et al., 2023a, 2023b). A search for meaning and for the role of oneself in the world is crucial during adolescence, particularly at the end of upper secondary school when students face critical decisions for their future. Thus, students who feel there is meaning in what they do and what happens to them develop an optimistic vision of the world, whereas those who feel that there is no meaning in their lives develop a pessimistic vision of the world. The association between Competence and Engagement might be the result of a school culture focusing on competition, so that if students do not feel competent, their engagement decreases. But also the tendency of young people to engage only in activities where they feel competent. Adults, on the contrary, take into greater consideration rules and responsibilities and tend to engage in activities even when they do not feel competent (but they might feel it is their responsibility). Thus, this disentanglement between competence and engagement might be interpreted as the adolescent process that is still ongoing. The above speculations should be specifically examined in empirical settings comparing adolescents and young adults. Further studies should explore the association between Competence/Engagement and Meaning/Optimism dimensions using more diverse and objective outcomes to assess unique predictive differentials, that we were not able to perform (and we address this as a limitation of the study). Nonetheless, the differential patterns of association with psychological correlates discussed above seem to suggest that they are distinct factors that might shape during late adolescence. In conclusion, we tested the factorial structure and validity of the WB-Pro in a sample of upper school students from Sardinia, thus generalizability of our results is somehow limited. Although our results are promising in supporting the use of the WB-Pro in adolescents, future studies should involve more diverse samples of young people.

## 6 Study 2: development of a learning path to enhance wellbeing in secondary school students and its applications

In the development of our learning path to improve wellbeing in school, first, we decided which dimensions of wellbeing included in WB-Pro to focus on. Literature review based on umbrella review (Iasiello et al., 2024), meta-synthesis (Carrillo et al., 2021), network analysis (Höltge et al., 2023; Stochl et al., 2019), and cross-cultural studies (Zambelli et al., 2025) showed that some dimensions (i.e., engagement, competence, optimism, meaning, positive emotions, self-esteem) recur as relevant aspects of wellbeing as well as critical life skills that schools should cultivate to help the global development of youths (WHO - World Health Organization, 1997). Moreover, in a canonical correlation analysis these dimensions appeared to be more relevant than other aspects of wellbeing^2^ according to an Italian adult sample (Scalas et al., 2023b). For these reasons, we focused primarily on them in developing the learning path and its associated activities.

To define the psychological activities associated with the chosen wellbeing dimensions, we conducted a review of primary studies (RCT or quasi-experimental design), focusing on upper secondary school interventions developed in the last 15 years. Programs had to be delivered to students in school settings (e.g., in the classroom; within school hours). They had to involve the dimensions of wellbeing we chose to focus on, and they had to provide a clear description of the training and the results of the interventions. Thus, to develop our training, we took into account the theoretical and applied issues, along with the activities, described in six studies that meet our criteria of relevance (Burckhardt et al., 2015, 2016; Freire et al., 2018; Perkins et al., 2021; Putwain et al., 2019; Veltro et al., 2015). In Supplementary Table S5 we report additional information on the activities developed for the program and their interconnection with activities developed in relevant scientific literature.

In relation to organizational aspects such as duration of the intervention, the literature showed heterogeneity of wellbeing interventions in school, with no specific association between duration and effectiveness (Gunawardena et al., 2023). Therefore, after consulting with the Principals of the schools contacted for the implementation of the program, we decided to develop short meetings (i.e., 60 min).

We used a protocol to develop the program (see Supplementary Table S6) to enhance wellbeing named “Take care of yourself”. To favor replicability of the intervention, our training program with proposed activities to enhance wellbeing is reported in Table 4. The training program consisted of six sessions of 60 min (every 7–10 days), each conducted by a psychologist with great experience in school-based interventions, focusing on one primary dimension of wellbeing (positive emotions, competence, engagement, meaning, optimism, self-esteem) and some additional dimensions relevant to the adolescence phase (positive relations, prosocial behavior, self-acceptance). Each session was structured around four phases (Borgato, 2016; Guarguaglini, 2007; Kaneklin, 2010; Quaglino, 2014): opening, activation, closing, and prescription (see Table 4 and Supplementary Table S6). Opening refers to the definition of the session’s framework; in the activation phase, the proposed activities help participants to move from practical experience to theoretical constructs; the closure phase consists of debriefing; finally, in the prescription phase, simple homework is assigned to be discussed at the beginning of the subsequent session. During the first meeting, participants received a notebook for recording information and completing the proposed activities in and outside the class.

**Table 4:** Synthesis of the characteristics of the learning path to enhance wellbeing in school.

Session	Primary dimension	Items of the WB-Pro related to the dimension	Classroom activities	Methodology used in each session	Activities between sessions
1	POSITIVE EMOTIONS	I generally feel cheerful. I am happy most of the time. All things considered, I would describe myself as a happy person.	Assignment: Identify and write down in your notebook five things you are grateful for and five good things in your life. Then share them with your group/classmates.	Interactive lecture. Individual work. Pair work. Group plenary session. Out-of-class assignments.	Assignment: “From today until our next meeting, practice five acts of kindness. How did they make you feel? Write them down in your notebook.”
2	COMPETENCE	I am competent and capable in the activities that are important to me. Most things I do, I do well. I am able to perform well and be successful in most things that I do.	Assignment: Identify and write down three strengths in your notebook. Then compare them with your partner.	Opening and discussion on session tasks. Interactive lecture Individual work. Work in pairs. Group plenary session. Guidelines for out-of-class assignments.	Assignment: “Ask two people you trust to list three strengths they see in you and write them down in your notebook.”
3	ENGAGEMENT	Most of the time I am really interested in what I am doing. I am almost always engaged and interested in my daily activities. I feel excited by many of the things I do.	First part—video projection on the theme of commitment. Second part—Work in pairs—identify and share at least three daily activities that you do with interest and involvement.	Opening and discussion on session tasks. Interactive lecture. Video screening. Individual work. Work in pairs. Group plenary session. Guidelines for out-of-class assignments.	Assignment: “Choose at least one activity to pursue with dedication and commitment, and write it down in your notebook.”
4	MEANING	I lead a purposeful and meaningful life. I feel I have a sense of direction in my life. My life has a clear sense of purpose.	Assignment: (divided into two subgroups) define what values are and identify at least five of the most important ones in life. Assignment: as a single group, address the topic of values, choose a single definition of value, combine the values identified in the two subgroups, and draw up a single list of values.	Opening and discussion on session tasks. Interactive lecture. Work in subgroups. Focus groups. Guidelines for out-of-class assignments.	Assignment: “Rank the values that emerged in class in order of importance. With regard to the first one, indicate which actions in your daily life show that it deserves first place among your values.”
5	OPTIMISM	I feel very optimistic about my future. My future looks very bright to me. I am always optimistic about my future.	Assignment, in pairs—a friend will meet you in two years’ time. Describe yourself, portraying the best and most realistic version of yourself that you can imagine right now.	Opening and discussion on session tasks. Interactive lecture. Individual work. Work in pairs. Group plenary session. Guidelines for out-of-class assignments.	Assignment: “For the next meeting, think of something wonderful that you would like to happen within the year. How does this thought make you feel?” Write it down in your notebook.
6	SELF-ESTEEM	I feel that I’m a person of worth. A lot of things about me are good. I feel that I have a number of good qualities.	First part– video screening, extract from a self-esteem movie. Second part– plenary session, summary of the material that emerged, ideas on the definition of self-esteem and self-acceptance. Closing activity: “Create your own personal motto!” Write it down in your notebook; take a photo of it with your cell phone…open the photo whenever you want to remember who you are and how much you are worth.	Opening and discussion on session tasks. Interactive lecture. Classroom screening. Group discussion. Individual work.	Try to practice everything you have learned in this course!

Before and after the program, we have conducted two additional group sessions to collect data on wellbeing (see Supplementary Figure S2). A meeting with two teachers, delegates of the school and present during the sessions, followed the six sessions. Finally, a follow-up session aimed at collecting additional information on wellbeing and students’ feedback on the experienced program concluded the training.

### 6.1 Method

We first tested the learning path in a pilot study (see Supplementary material) and subsequently revised and administered it to a larger sample.

#### 6.1.1 Participants and procedures

**Figure 1:**
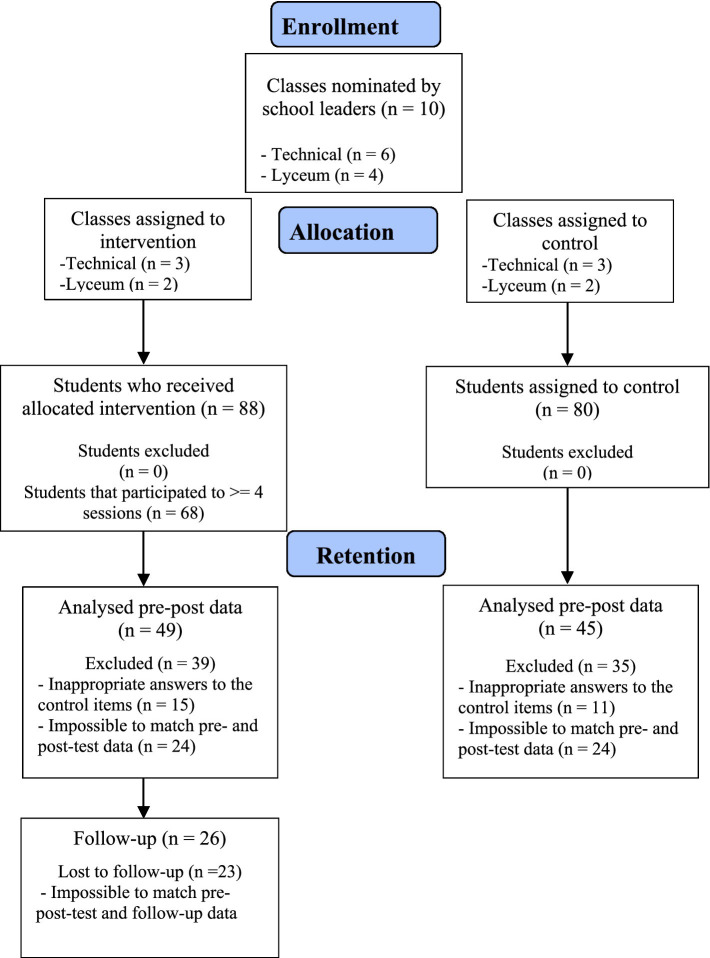
Flowchart of participants for the intervention study.

Students of 12 classes of the final year of urban upper secondary schools from Sardinia (Italy) were involved in the study; respectively two for the pilot study (intervention, *N* = 15; control, *N* = 15); and 10 for the intervention study (intervention, *N* = 88; control, *N* = 80). Supplementary Figure S3 and Figure 1 represent the participants’ flow for the pilot and intervention study, respectively. In Supplementary Table S7 it is reported the Intraclass Correlation Coefficient.

The attrition was high (see Table 5); some students did not answer properly to the control items (e.g., “To this question, please respond 1,” see Study 1), and for some students it was not possible to match the pre- and post-test due to absence on one of the data collection sessions (see Supplementary Table S1).

**Table 5:** Sample after attrition.

Study	Group	*N*	Male	Age mean	Age s.d.
Pilot	Intervention	14	13	18.14	0.66
	Control	11	11	17.82	0.40
Intervention	Intervention	49	33	19.2	0.69
	Control	45	24	19.20	0.56

The study was approved by the Ethics Committee of the University of the authors. We also obtained approval from the School Committee and collected informed consent from parents and students. Safeguarding procedures were followed with a preliminary debriefing with the school Principal, identification of at least one delegate from the school, written communication to the parents to explain the general aim of the study, to get explicit parental approval with opt-in procedures, and to gain informed consent (signed by both parents for underage students).

In technical upper secondary schools, there is a prevalence of males, whereas in lyceum, there is a prevalence of females. Participants were all Caucasian and of intermediate socio-economic level. Within the same school, due to organizational issues of the school and availability of teachers in providing their hours for the program, it was not possible to randomly assign the classes to the intervention and control groups. However, classes were similar for socio-economic level, number of BES, and so on (it should be noted that, in the Italian educational system, specific criteria are used to form classes with similar conditions).

The pilot study groups completed the battery of questionnaires at the end of September 2024, before the beginning of the program, and at the end of November 2024, after the end of the program. The intervention study was conducted at the beginning of January 2025 and at the end of April/beginning of May 2025. Finally, in May 2025, we conducted the follow-up sessions. Results of the pilot study are discussed in Supplementary material.

#### 6.1.2 Implementation and roles

The training protocol based on the identified variables and activities was developed by the research team, with the special contribution of a psychologist with extensive experience in school-based interventions, who also delivered the training (see Supplementary Table S6). Before and after each session, there was a debriefing with the research team supervisor to monitor the fidelity of the intervention. In each school, teachers delegated by the school were present during the sessions and took part in the activities. Curricular teachers were also welcomed as active members of the class group.

The program requires different training for teachers depending on their expertise. The teachers with psycho-pedagogical competencies or school psychologist training would require simply a few hours of debriefing. The training of other teachers would require 2/3 h of training for each session before program delivery, and 3 h of supervision during delivery (1 h after the first meeting, 1 h after the third meeting, 1 h after the last meeting). Optional in-class modeling for Session 1. Sessions can be delivered as part of wellbeing/PDHPE (Personal Development, Health and Physical Education) periods, when presents. Resources available to the delivery personnel are slides and a fidelity checklist. In terms of feasibility, 77% of the students participated in at least 4 out of 6 meetings. Each meeting was designed for a duration of 60 min. However, since the delivery occurs during school hours, a few delays might occur between classes. Thus, we believe that a timetable fit would require at least 50 min for each meeting. For the present intervention timetable fit was good with delays around 5/10 min max.

As part of the collaborative consultation with one of the schools that was particularly sensitive to the issue of students’ wellbeing, we offered the school delegate teachers the training on our wellbeing program, to favor autonomy and independence of the school in the process of enhancing good practices for students’ wellbeing and enhancing teachers’ self-efficacy and high-quality professional development.

#### 6.1.3 Intervention fidelity

Attention was given to intervention fidelity to allow replicability and to ensure that the observed trends were due to the program itself and not to variations in implementation (Barnett et al., 2014). In Supplementary material can be found information about the intervention fidelity (see Supplementary Table S8), the fidelity checklist (Supplementary Table S9) that we developed to monitor the intervention, and the Satisfaction with activities questionnaire for participants (Supplementary Table S10).

#### 6.1.4 Instruments

To collect information about wellbeing, we administered a battery of instruments before and after the program. According to the aim of the interconnection between the intervention and its measurement, we will discuss responses to the WB-Pro and SWLS (see Study 1 for a description). Moreover, a satisfaction questionnaire for the activities was carried out concerning various aspects, such as interest and usefulness (e.g., “Overall, do you think it was helpful to participate in the proposed activities?”) of the program activities and organizational aspects (“Was the overall duration of the program adequate?”). Finally, for the follow-up, we used the WB-Pro Short version (15 items).

#### 6.1.5 Design and plan of analysis

Allocation occurred at the class level; thus, the class constituted the unit of assignment. The intervention study comprised 10 classes (5 intervention; 5 control—see Figure 1). To account for the non-independence of observations within classes, outcomes were analyzed using linear mixed-effects models including a random intercept for class. Fixed effects included condition (intervention vs. control), time (pre-test, post-test), and the time × condition interaction. Baseline scores were included as covariates (ANCOVA specification) to adjust for pre-existing differences between groups. Models were estimated using restricted maximum likelihood (REML), and degrees of freedom were calculated using the Satterthwaite approximation to provide a small-sample correction appropriate for clustered designs. Because allocation occurred at the class level, the estimated intervention effect represents a between-class contrast adjusted for baseline rather than an individually randomized causal effect.

Mixed-model likelihood handled missing outcomes under a missing-at-random (MAR) assumption; observations missing covariates were excluded for the relevant analysis. Pre-test ICCs for primary outcomes ranged between 0.04 and 0.17, supporting the use of mixed models (see Supplementary Table S7). The contribution of the predictors was measured with marginal R^2^ coefficients of determination. This coefficient in a GLMM quantifies the variance explained by all fixed effects of the model (Nakagawa and Schielzeth, 2013). Primary contrasts are reported as Hedges’ g with 95% confidence intervals. Univariate follow-ups are considered exploratory and reported with effect sizes and unadjusted *p* values. Descriptive statistics were computed for the Satisfaction questionnaire. We assessed groups’ equivalence for the pre-test measures with a MANOVA (Wilks’ Lambda = 0.83; *F*_(9, 83)_ = 1.93, *p* = 0.058; *η*^2^ = 0.173). Overall, the control group reports mean levels higher than the intervention group at the pre-test measures; however, in many cases these differences are not statistically significant (see Supplementary Table S11). These values are in line with ICCs reported in Supplementary Table S7 and reflect the non-randomization of classes. Even though some dimensions showed moderate clustering, inclusion of the class-level random intercept ensures that standard errors and statistical inferences appropriately reflect the hierarchical structure of the data. Given class-level allocation, the treatment effect represents a between-class contrast adjusted for baseline, and inference is therefore at the class level.

### 6.2 Results

**Figure 2:**
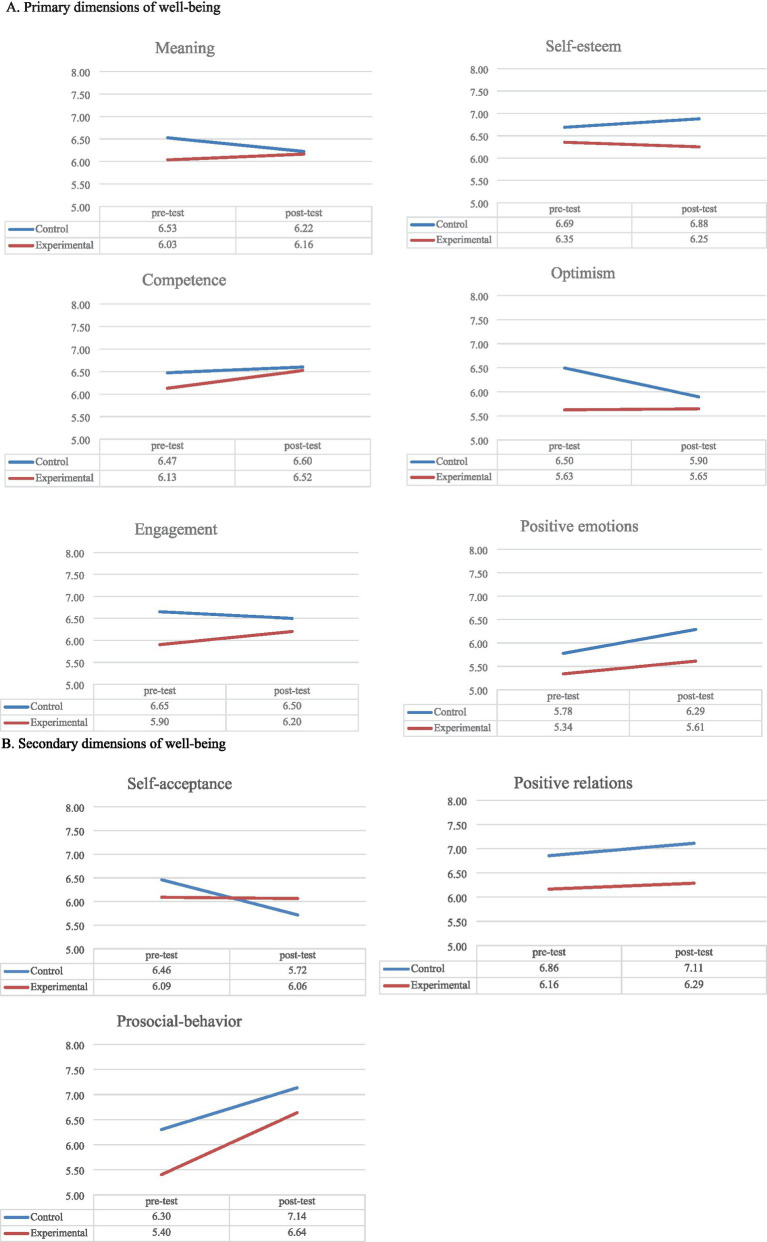
Means over time for the primary and secondary dimensions of the wellbeing training in the intervention and control groups: **(A)** Primary dimensions of wellbeing; **(B)** Secondary dimensions of wellbeing.

***Quantitative Results***. Mixed models showed small fixed effect in terms of explained variance (*R*^2^) of the interaction between the pre-test measure and group (intervention-control) for Self-acceptance (*F*_(1, 84)_ = 5.64, *p* < 0.05; *R*^2^ = 0.219; Hedges’ *g* = 0.33, 95% CI [−0.07, 0.74]) and Self-esteem (*F*_(1, 89)_ = 3.86, *p* = 0.053, *R*^2^ = 0.230; Hedges’ *g* = −0.34, 95% CI [−0.74, 0.07]), and a moderate effect in relation to Meaning (*F*_(1, 90)_ = 3.68, *p* = 0.058, *R*^2^ = 0.365; Hedges’ *g* = 0.16, 95% CI [−0.24, 0.56]), regardless statistical significance. In relation to Meaning, a primary dimension of our intervention to enhance wellbeing, a decrement from pre- to post-intervention affected the control group, whereas the values remained almost stable for the intervention group (with a slight increment; see Figure 2). During the training, activities promoting the search for meaning were proposed. These activities might have resulted in a protective trend in the intervention group, helping students deal with anxiety associated with the final exam (and what comes next, with its uncertainties), which seems to have affected the control group, instead. A similar process might have happened for Self-acceptance (see Figure 2), which was a secondary dimension in our training. For Self-esteem (primary dimension), a negative effect emerged with a slight increase for the control group and a slight decrement for the intervention group (see Figure 2); this might be the result of an increase in self-consciousness due to the training (e.g., Reitz, 2022).

Concerning other dimensions of the wellbeing training, results showed small to moderate main effects related to pre-test measures. For primary dimensions of Competence (*F*_(1, 88)_ = 29.87, *p* < 0.001, *R*^2^ = 0.263) and Positive emotions (*F*_(1, 82)_ = 11.05, *p* < 0.001, *R*^2^ = 0.145), and for secondary dimensions of Prosocial behavior (*F*_(1, 88)_ = 8.50, *p* < 0.01, *R*^2^ = 0.114) and Positive relations (*F*_(1, 83)_ = 31.09, *p* < 0.001, *R*^2^ = 0.312), post-test measures seem to increase for both groups. Even though no statistically significant interaction effect emerged, Figure 2 shows that for Engagement (primary dimension; *F*_(1, 88)_ = 32.23, *p* < 0.001, *R*^2^ = 0.275), the increment at post-test appeared associated with the intervention group; whereas for Optimism (primary dimension; *F*_(1, 90)_ = 53.16, *p* < 0.001, *R*^2^ = 0.370), the decrement at post-test appeared only for the control group (see Figure 2). Detailed information about means and standard deviations is reported in Supplementary Table S11, whereas in Supplementary Table S12, we report marginal means with 95% confidence intervals.

**Figure 3:**
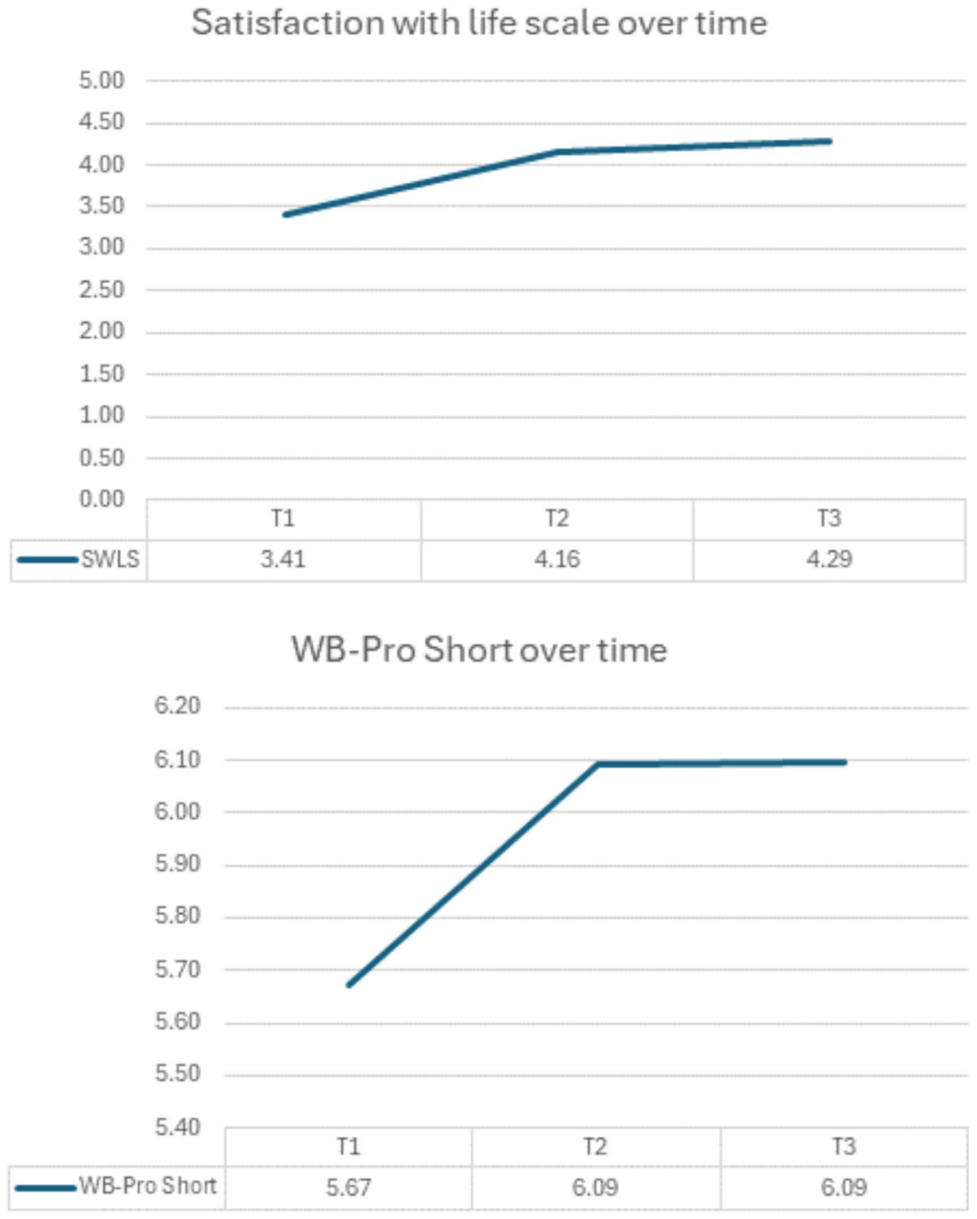
Wellbeing over time in the intervention group of the intervention study. T1 = pre-test; T2 = post-test; T3 = follow-up.

In relation to the follow-up evaluation, an exploratory repeated measure ANOVA in relation to within subjects showed a small effect (Wilks’ Lambda = 0.64; *F*_(4, 90)_ = 5.69, *p* = 0.001; *η*^2^ = 0.202). At the univariate level, a moderate effect emerged for SWLS (*F*_(2, 46)_ = 11.40, *p* = 0.001; *η*^2^ = 0.331) and a weak one for WB-Pro Short version (*F*_(2, 46)_ = 4.29, *p* = 0.02; *η*^2^ = 0.157). As can be observed in Figure 3, the intervention resulted in a maintained improvement on selected measures. Nonetheless, these results should be interpreted cautiously, due to the exploratory nature of the study.

***Qualitative results***. In relation to students’ feedback, the mean values at the Satisfaction questionnaire, were higher than the midpoint of the answer response scale for all items, even though they were lower than the pilot sample (*N* = 77, mean = 6.55, s.d. = 2.25). Participants found the proposed activities useful (*N* = 77, mean = 6.29, s.d. = 2.28) and judged the conductor supportive (*N* = 77, mean = 6.57, s.d. = 2.27). It should be noted that statistically significant differences emerged between the two schools involved in the study, with higher levels of satisfaction for the technical school (see Supplementary Table S10).^3^ Concerning organizational aspects, the 51% participants (*N* = 36) found the training adequate in terms of length, for the 43% participants (*N* = 33) it was too short, and for the 6% (*N* = 5) it was too long. The 73% of participants (*N* = 56) found the duration of each meeting adequate, whereas for the 17% (*N* = 13) the meetings were too distant in time, and for the 10% (*N* = 8), they were too close in time. In relation to the open-ended part of the questionnaire, participants referred to the opportunity to reflect and think, the involvement, and cooperation of all participants as strengths of the program; whereas among the weaknesses, they more frequently addressed that the training was too short. Interviews with two teachers confirmed the general interest and satisfaction of students in relation to the program.

### 6.3 Discussion of study 2

In study 2, we described the development of our program to enhance key dimensions of wellbeing and to provide students with psychological resources during a particularly demanding phase of secondary education.

We applied it in a pilot sample to better understand under which circumstances the intervention could be successfully delivered (Gadke et al., 2021) and, after minimal adaptation based on this first application and feedback from students and school delegates, we tested it in a different larger sample. Intervention and control groups were involved in both samples, and classes were matched within each school; we used a pre-post intervention design, without randomization, to comply with school organizational needs.

In terms of outcomes measures, the pilot study did not show substantial differences in wellbeing at the pre- and post-test highlighting a weakness in sensitivity to change; however, the small sample size might have affected these results Students’ satisfaction questionnaire and interviews with school delegates showed an adequate acceptability of the intervention.

In the intervention study, results of mixed models that account for the clustering effect suggest a potential protective trend for our program to enhance wellbeing. Data seem to confirm that the final upper secondary school year is complex for adolescents (de Moor and Christiaens, 2024), particularly the last months as the examination approaches; nonetheless, whereas in the control group emerged a decrement for meaning and self-acceptance presumably related to worries about the final examination and what comes next (e.g., preoccupation about own adequacy in meeting own and/or other aspirations and doubts about one’s path in the world), in the intervention group on the contrary the values remained stable suggesting that the proposed activities might have strengthen the ability of students to face this kind of anxieties helping them in keeping a positive state of mind. In relation to self-esteem, a slight decrement was found for the intervention group and a slight increment for the control group. These results might be associated with instability of this variable during adolescence and contingency (e.g., an increase in self-consciousness due to the training) (e.g., Reitz, 2022). Finally, responses to the WB-Pro Short version and the SWLS suggested an increase in wellbeing from pre- to post-intervention, which remained stable after 1 month of the intervention. Therefore, although improvements were not uniform, the relative stability in the intervention group, compared with the decline in the control group, is consistent with a protective effect of the program during a demanding school period. Nonetheless, considering the quasi-experimental, non-randomized class design of our study and the explorative nature of the follow-up, our results should be interpreted with caution, and further studies are needed to test the usefulness of our training to enhance wellbeing in upper secondary school students.

Thus, our results are promising, but they are not free from critical issues concerning feasibility (Gadke et al., 2021), with limitations in terms of research design, attrition and practicality. When we first designed the study, we intended to randomize classes to control and intervention groups. However, no school was available to comply with this request. Therefore, to conduct the study, it was necessary to make compromises and accept that comparison and intervention classes would be assigned based on the curricular teachers’ availability to volunteer their hours for the intervention. Allocation was therefore quasi-experimental and conducted at the class level. The study included 10 classes in total (5 intervention; 5 control), and although mixed-effects models with random intercepts were used to account for clustering, statistical inference regarding the intervention effect is driven by the number of clusters rather than the number of individual participants. Consequently, effect estimates and associated confidence intervals should be interpreted as preliminary feasibility evidence rather than definitive tests of efficacy. In addition, because the control group reported somewhat higher baseline levels across several dimensions, even after statistical adjustment for pre-test scores, residual effects associated with baseline imbalance cannot be entirely ruled out. Future studies with a randomized class design are necessary to support conclusions.

Moreover, we experienced high levels of attrition; some students were present only on one measurement session (pre-test, post-test, follow-up), and thus it was not possible to match their results to test the effects of the intervention. Some students did not answer correctly to the control items in the survey (e.g., “To this question, please respond 1”), maybe due to the length of our survey. Since it was not possible to distinguish between distraction and non-collaboration, we decided to eliminate all students with wrong answers to the control items. For these reasons, future research should be advised on the necessity to start with larger samples.

Some limitations refer specifically to organizational aspects of our program. First, each meeting was conducted during class time; thus, it often resulted in shorter than 60 min (although never less than 50 min), due to a change of curricular teacher, or delays of students in the first hour of the school day. Second, curricular teachers were not always supportive of the program; thus, sometimes, when they remained in class, it represented a distraction for students. In our research, this happened particularly with one school, where maybe teachers felt they had to comply with a requirement of the school principal, but did not really believe in the program. For future studies, more attention should be given to engaging both students and teachers in the program. Teachers’ motivation is in fact a key aspect for their behavior and an instruction of quality (Lazarides et al., 2025). To develop the sense of autonomy and meaningful engagement of teachers in the process of enhancing good practices for students’ wellbeing, we offered, in exploratory way, training on our wellbeing program, to enhance teachers’ self-efficacy and high-quality professional development; this represents a relevant step to allow delivery of the program by community providers (Onken et al., 2014).

## 7 General discussion

To ensure coherence between the development of an intervention program and the choice of the measurement instrument, in this research, we have validated the WB-Pro in students of upper secondary school, and, moving from some of its scales relevant for adolescents, we have developed a multidimensional program to enhance wellbeing. The need for this kind of intervention has often been highlighted in the literature for several reasons. For example, the final years of secondary school are potentially stressful for students since they must face important changes at the social and personal level (e.g., de Moor and Christiaens, 2024), which might negatively affect academic motivation and performance (Galton et al., 2002), and school and global wellbeing (de Moor and Christiaens, 2024), producing an increment of internalizing and externalizing problems (Nielsen et al., 2017; Puja et al., 2024). Wellbeing is a complex and multidimensional construct that has been studied from different perspectives (e.g., Herd, 2022; Iasiello et al., 2024). In recent years, several authors have suggested the need to integrate these perspectives from theoretical and practical points of view (e.g., Herd, 2022; Keyes and Waterman, 2003; Lent and Brown, 2008; Marsh et al., 2020; Seligman, 2011). Although wellbeing has a long history in the adult population, research on adolescents’ wellbeing is still limited (Mahajan et al., 2023), as well as its theoretical understanding (Avedissian and Alayan, 2021). Moreover, from an applied perspective, current instruments for assessing psychological wellbeing in upper secondary school are limited in their ability to capture its multifaceted nature.

For these reasons, we conducted two studies. The first aimed at adapting to upper secondary school students, the WB-Pro (Marsh et al., 2020; Scalas et al., 2023a, 2023b), a recent multidimensional instrument moving from the conception of wellbeing as positive mental health and embracing hedonic and eudaimonic aspects of wellbeing. The second study aimed at the development and application of a multidimensional program to enhance wellbeing in upper secondary school students. Responses of the participants to the WB-Pro (and to the SWLS) have been used to evaluate the effects of our program on wellbeing enhancement from a multidimensional perspective, through a quasi-experimental, non-randomized class design.

Study 1 showed that the WB-Pro is a valid and reliable instrument to evaluate wellbeing from a multidimensional perspective in upper secondary school students. It has the advantage of producing an individualized profile that can be used to tailor interventions and, in case of a shortage of time, two formative short versions can be used. Nonetheless, some specificities associated with upper secondary school students emerged that should be better examined in future research.

Study 2 might suggest protective trends for some dimensions of our learning path to enhance wellbeing, particularly in helping upper secondary school students to deal with the difficulties of their final year of school, characterized by worries and uncertainty associated with the exam and what comes next. Moreover, the intervention group seems to show maintained improvement after 1 month of the intervention on selected measures (WB-Pro Short version, SWLS). However, since allocation was quasi-experimental and conducted at the class level (with statistical inference driven by the number of clusters), results should be interpreted as preliminary feasibility evidence rather than definitive tests of efficacy. Finally, the students generally appreciated the training and were satisfied with it and its organization. Nonetheless, additional qualitative information on participant’s experience might have been useful to understand the mechanisms underlying some of our results; thus, future research would benefit from a mixed-methods design. Moreover, the application of a detailed framework to assess feasibility of the intervention could maximize its internal and external validity (Gadke et al., 2021).

In relation to the full cycle of intervention research and considering the multi-stage model by Onken et al. (2014), the intervention to enhance wellbeing of upper-school students we propose here cannot be considered completed. The program “Take care of yourself” descends and is informed by literature on wellbeing and adolescence (stage 0); the activities proposed are connected to specific dimensions of wellbeing and can be measured properly with the WB-Pro (Marsh et al., 2020); also, materials have been developed to document the program (stage I); the program has been delivered (in real-world) by a research provider, it has been adapted to school settings and tested in a pilot study, revised and tested again (stage II). Nonetheless, more needs to be done, the program needs to be tested with community providers and maybe adjusted; (stage III), generalized to more diverse samples (stage IV), to finalize a program based on scientific evidence that can be disseminated and implemented into community settings (stage V).

## 8 Conclusion

In conclusion, the final years of upper secondary school are a critical period since students are called to deal with important changes in relation to intrapsychic and interpersonal factors (de Moor and Christiaens, 2024) that might negatively affect psychological wellbeing and adaptation (Galton et al., 2002; Nielsen et al., 2017; Puja et al., 2024). Favoring psychological wellbeing during these years is important to facilitate this developmental process. Thus, it is important to have reliable and valid instruments to evaluate wellbeing from a multidimensional point of view and to develop programs to enhance wellbeing. In our research, we validated the WB-Pro (Marsh et al., 2020; Scalas et al., 2023a, 2023b) in upper secondary school students and developed a feasible class-time program to enhance wellbeing connected to some of its dimensions most relevant in adolescence. Typical delivery is six 60-min sessions (≥50 min workable), plus two 30-min sessions to evaluate wellbeing at pre- and post-intervention. We observed small, protective trends (e.g., meaning, optimism), with high acceptability, and ≥4/6 session dose in 77% of students. Schools can implement using existing staff, with training and consultative supervision from a school psychologist, and a fidelity checklist (aim ≥ 80%). Even though the training needs to be applied and tested in a larger and more diverse sample of upper secondary school students, our results are promising and seem to highlight protective trends with maintained improvement on selected measures for our wellbeing multidimensional program.

## Data Availability

The raw data supporting the conclusions of this article will be made available by the authors without undue reservation.

## Footnotes

1 These two routes are quite common in Italy. The Italian secondary education system lasts 8 years and is organized in two stages: middle school (ages 11–14) and high school (ages 14–19). There are three types of high school: lyceum (the most popular with about the 55% of enrolment; aimed at preparing students for university); technical institutes (32% of enrolment: aimed at preparing students for both work and university), and institutes for specific professions (13% of enrolment; including practical work related to industry).

2 In the subsample of 383 students of study 1, we performed a canonical correlation analysis including all WB-Pro dimensions and correlates and we found results similar to the adult sample of Author. Indeed, the dimensions with the highest canonical loadings (and thus the most relevant for wellbeing) are mostly overlapping with those found in adults; to those positive relations can be added, which is known to be a critical dimension during adolescence (see Supplementary Tables S4a–c).

3 It should be noted that a different attitude toward the research project appeared in the schools involved in the study. The school delegates of one school were more engaged and interested in the project and were probably more capable of involving their colleagues and students.
