# Supplementary material for: Well-being in upper secondary school students: confirming a 15-factor structure and a class-allocated feasibility trial with practice guidance
Source: Front Psychol. 2026 Mar 19;17:1790035. doi: 10.3389/fpsyg.2026.1790035 (PMC13044007; doi:10.3389/fpsyg.2026.1790035)
Supplement: Supplementary file 1 [file Data_Sheet_1.pdf]

Supplementary Material

Supplementary Material

Well-Being in Upper Secondary Schools Students: Confirming a 15-Factor Structure and a Class-Allocated Feasibility Trial with Practice Guidance

Scalas, Pedditzi, Cuccu, Fadda, Marsh

1 Additional information on the pilot study results

Aim of the pilot study

Principal aims of the pilot study were to understand: 1) if the training was feasible within the school context; 2) if the proposed activities were accepted by upper-school students; 3) the level of satisfaction with the training, particularly in relation to the proposed activities.

Plan of analysis

Concerning the pilot study, we tested in an exploratory way the equivalence of the intervention and control groups in the pre-test WB-Pro dimensions and SWLS with independent t-tests and the changes for both groups, after the program with paired t-tests.

Results

Quantitative results

Results of t-tests for independent groups (under the assumption of homogeneity of variance, see Table S13), showed no difference across groups at the pre-test, except the Resilience scale, which favored the control group (see Table S14). This difference no longer seems to exist after the training (see Table S14). Moreover, no statistically significant change in well-being dimensions for the intervention group (nor for the control group) appeared at post-test (see Table S15), but this could be the result of the small sample size of participants involved in the pilot study.

Qualitative results

The satisfaction questionnaire. The satisfaction questionnaire revealed general satisfaction with the program (N =13; mean = 8.46, s.d. = 1.81). Participants found the proposed activities interesting (N =13; mean = 8.31, s.d. = 1.03) and useful (N =13; mean = 7.46, s.d. = 2.60), and judged the conductor supportive (N =13; mean = 7.62, s.d. = 1.98). Concerning organizational aspects, nine participants (out of 13 respondents) found the meetings too short, and most of them would have liked additional meetings (11 out of 13). Participants noted that the program helped them to value themselves as people worthy of value, to experience positive emotions, to enhance their well-being, and to value what they do. In the open-ended section of the questionnaire, one participant expressed personal difficulties in expressing him/her-self in front of others.

Notes from the research provider of the training. The conductor of the program highlighted a few practicality issues. The first concerns time management within the school schedule. Since the session

was scheduled during school hours, it fell between the schedules of one teacher and another, which inevitably reduced the session's net time; moreover, the first and last school hours were critical since some students arrived late or had to leave early to catch trains of buses. A second critical issue concerned the level of collaboration of the teacher present in the classroom at the time of the intervention. The levels and methods varied greatly, and this aspect resulted in some variability in classroom management. Finally, it should be noted that the decision to involve the entire class in the group discussions may have made some participants feel "forced" to participate.

Notes from the school delegates. Participants perceived the intervention as appropriate and potentially effective. The teachers, who were school delegates and attended the meetings regularly, noted the general interest and curiosity of students in relation to the program, and that some students asked if it would be possible to transform the program into a curricular activity to deliver once a week during the school year. An aspect particularly appreciated by the participants was the activity notebook that was given to participants on the first day to write down notes and the results of the paired, group, and individual activities carried out in class and at home.

Conclusion

In conclusion, the pilot study revealed that the program was welcomed by the students, who valued it positively. No substantial mean change emerged in well-being dimensions specific to the training (but in relation to resilience, the initial difference at the pre-test, favoring the control group, vanished in the post-test) highlighting a weakness in sensitivity to change. This might be the result of the small sample size involved in the pilot study. On the basis of feedback from students, teachers and the research provider, a few changes in procedural aspects of the program were made. 1) The first and last school hours were avoided; 2) We decided to stress to the curricular teachers that they were not obligated to stay in class during the program meetings, but that if they stayed in class, they needed to actively participate as members of the group and not just observers. 3) Instead of strictly following the criterion of proximity, students were left free to choose their partner/s in couple/small group activities.

2 Additional information about the schools involved in the intervention study

The Italian secondary education system lasts eight years and is organized in two stages: middle school (ages 11–14) and high school (ages 14–19). There are three types of high school: lyceum (the most popular with about the 55% of enrolment; aimed at preparing students for university); technical institutes (32% of enrolment: aimed at preparing students for both work and university), and institutes for specific professions (13% of enrolment; including practical work related to industry).

Within the regional context of Sardinia, the technical institute involved in the study is considered medium level based on the ESCS (Economic, Social, and Cultural Status) index, with a student population from a diverse background, typical of a large technical institute serving a large area (hinterland of Cagliari). Despite its non-elite socioeconomic background, the institute achieves excellent results: Eduscopio data regularly recognizes this institution for the quality of its technical training and the high employability rate of its graduates. Results in the national INVALSI tests are often above the regional average for English, but below average for Maths, and Italian.

Regarding the pedagogical Lyceum involved in the study, the socioeconomic status of its students is classified as medium within the regional context, with a relevant number of foreign students. A relevant number of NEET is associated to this school. Results in the national INVALSI tests are often below the regional average for Maths, Italian, and English.

3 Supplementary Figures and Tables

Table S1. Distribution of control questions in study 1 and study 2

**STUDY 1**										
	Response-scale options	Item 1	Item 2	Item 3	Item 4	Item 5	Item 6	Item 7	Item 8	Item 9
Sample 1	1	8	9	9	403	7	5	5	8	4
	2	1	1	0	6	2	1	4	6	409
	3	0	4	3	4	3	404	0	0	2
	4	4	2	1	2	2	5	408	3	3
	5	3	1	1	1	4	1	2	4	4
	6	3	1	407	3	5	7	4	4	3
	7	4	1	8	4	406	4	4	2	3
	8	408	3	3	2	5	4	2	2	1
	9	2	407	3	4	1	0	4	2	4
	10	6	11	4	9	3	7	5	406	4
	missing	0	0	0	1	1	1	1	2	2
	Total	439	439	439	439	439	439	439	439	439
Sample 2	1	6	1	1	97	2	3			
	2	1	0	0	1	1	0			
	3	0	2	1	1	1	1			
	4	0	0	2	0	0	1			
	5	0	1	1	3	2	1			
	6	2	0	96	0	1	1			
	7	0	0	2	1	98	2			
	8	97	1	1	2	0	0			
	9	0	99	1	0	0	2			
	10	1	3	2	2	2	96			
	missing	0	0	0	0	0	0			
	Total	107	107	107	107	107	107			
**STUDY 2**										
	Response-scale options	Item 1	Item 2	Item 3	Item 4	Item 5	Item 6	Item 7	Item 8	Item 9
Post-test	1	3	2	3	112	2	2	2	2	3
	2	1	0	0	3	1	0	1	0	115
	3	0	0	1	2	1	114	1	1	1
	4	0	2	1	1	0	1	118	0	1
	5	2	3	2	2	3	1	0	3	3
	6	3	4	114	2	2	1	4	2	4
	7	2	2	3	4	117	2	0	3	0
	8	118	3	2	1	0	3	0	2	0
	9	0	112	1	1	1	0	0	2	1
	10	1	1	2	1	3	2	3	114	1
	missing	0	1	1	1	0	4	1	1	1
	Total	130	130	130	130	130	130	130	130	130

Pre-test	1	4	3	4	167	2	2	2	3	1
	2	0	0	0	1	1	0	0	1	166
	3	0	1	2	1	2	166	1	0	2
	4	1	1	0	0	1	3	164	3	1
	5	0	1	0	1	1	2	3	1	2
	6	0	0	167	2	2	1	3	2	1
	7	1	1	1	0	164	0	1	0	0
	8	168	2	0	0	1	0	1	1	2
	9	0	165	1	0	1	0	0	1	0
	10	3	2	2	5	2	3	1	165	2
missing		1	2	1	1	1	1	2	1	1
Total		178	178	178	178	178	178	178	178	178

Study1. The sample for study 1 was formed by 2 samples. In sample 1, 9 control questions were used; whereas in sample 2, for shortage of time, a shorted survey was used and only 6 control questions were included. The retained students had a similar age-mean in comparison to the excluded students in both samples (sample 1 respectively: age-mean= 17.90, d.s. = .69, age-mean= 17.72, d.s. = .68; sample 2 respectively: age-mean= 17.47, d.s. = .70, age-mean= 17.73, d.s. = .59). The distribution of school type was similar across the retained (Lyceum = 85%) and excluded (Lyceum = 80%) samples.

Study 2. The retained students had a similar age-mean in comparison to the excluded students (respectively: age-mean= 19.15, d.s. = .54, age-mean= 19.18, d.s. = .52). The distribution of school type was different across the retained (Lyceum = 37%) and excluded (Lyceum = 71% samples. Confirming the different attitude to the project in the two schools (see table S10).

Table S2. Information about skewness and kurtosis of WB-Pro items from study 1

	Min	Max	Mean	S.D.	Skewness	S.E.	Kurtosis	S.E.
WB1	1	9	6.33	2.42	-0.69	0.12	-0.51	0.25
WB2	1	9	7.03	2.03	-1.15	0.12	0.93	0.25
WB3	1	9	6.21	1.94	-0.68	0.12	0.18	0.25
WB4	1	9	5.71	2.10	-0.45	0.12	-0.45	0.25
WB5	1	9	7.50	1.43	-1.40	0.12	**2.56**	0.25
WB6	1	9	5.61	2.19	-0.34	0.12	-0.74	0.25
WB7	1	9	6.04	2.18	-0.63	0.12	-0.44	0.25
WB8	1	9	5.83	2.06	-0.57	0.12	-0.23	0.25
WB9	1	9	5.85	1.97	-0.58	0.12	-0.20	0.25
WB10	1	9	6.07	2.39	-0.61	0.12	-0.63	0.25
WB11	1	9	5.66	2.17	-0.38	0.12	-0.62	0.25
WB12	1	9	6.70	1.94	-1.00	0.12	0.66	0.25
WB13	1	9	6.21	1.85	-0.79	0.12	0.36	0.25
WB14	1	9	5.35	2.08	-0.28	0.12	-0.71	0.25
WB15	1	9	6.97	2.03	-1.12	0.12	0.72	0.25
WB16	1	9	5.78	2.12	-0.47	0.12	-0.46	0.25
WB17	1	9	6.32	1.80	-0.66	0.12	0.16	0.25
WB18	1	9	5.90	2.22	-0.57	0.12	-0.47	0.25
WB19	1	9	6.51	1.85	-0.80	0.12	0.30	0.25
WB20	1	9	6.36	2.07	-0.81	0.12	0.14	0.25
WB21	1	9	6.11	2.08	-0.60	0.12	-0.31	0.25
WB22	1	9	6.36	1.76	-0.83	0.12	0.59	0.25
WB23	1	9	6.89	1.89	-0.98	0.12	0.56	0.25
WB24	1	9	5.70	2.25	-0.45	0.12	-0.67	0.25
WB25	1	9	6.56	1.77	-0.94	0.12	0.77	0.25
WB26	1	9	7.60	1.82	-1.67	0.12	**2.79**	0.25
WB27	1	9	6.21	1.91	-0.77	0.12	0.24	0.25
WB28	1	9	8.01	1.47	-1.94	0.12	**4.36**	0.25
WB29	1	9	7.34	1.56	-1.26	0.13	**2.20**	0.25
WB30	1	9	5.61	2.13	-0.37	0.12	-0.52	0.25
WB31	3	9	7.64	1.27	-0.99	0.12	0.91	0.25
WB32	1	9	6.30	2.23	-0.77	0.12	-0.25	0.25
WB33	1	9	6.36	2.14	-0.73	0.12	-0.10	0.25
WB34	1	9	5.42	2.17	-0.25	0.12	-0.79	0.25
WB35	1	9	6.48	1.68	-0.78	0.12	0.47	0.25
WB36	1	9	6.02	2.32	-0.67	0.12	-0.46	0.25
WB37	2	9	7.46	1.43	-1.11	0.12	1.43	0.25
WB38	1	9	6.06	2.28	-0.64	0.12	-0.39	0.25
WB39	1	9	5.91	2.23	-0.47	0.12	-0.71	0.25
WB40	1	9	7.08	1.61	-0.88	0.12	0.83	0.25
WB41	1	9	6.77	2.03	-0.93	0.12	0.30	0.25
WB42	1	9	6.47	2.02	-0.81	0.12	0.04	0.25
WB43	1	9	5.53	2.30	-0.46	0.12	-0.65	0.25
WB44	1	9	5.03	2.55	-0.04	0.12	-1.13	0.25
WB45	1	9	6.00	2.20	-0.59	0.12	-0.42	0.25

WB46	1	9	6.12	2.26	-0.76	0.12	-0.25	0.25
WB47	1	9	6.85	1.69	-1.04	0.12	1.25	0.25
WB48	1	9	5.95	1.95	-0.47	0.12	-0.16	0.25
WB49	1	9	6.77	1.67	-0.88	0.12	0.78	0.25

Table S3. Fit indices of Confirmatory Factor Analysis of the 15-dimensional structure of the WB-Pro in an Italian sample of high-school students

	$\chi 2$	df	Scf	CFI	TLI	RMSEA
CFA	2018.597***	1021	1.22	.922	.910	.045
Bifactor-CFA	2498.712***	1078	1.22	.888	.878	.053

Note. * p< .001

In addition to classical CFA, we also tested Bifactor-CFA to explore the possibility of gaining an overall indicator of well-being. However, the bifactor model showed poor fit indices (see Table S1), suggesting that the bifactorial model is not an appropriate representation for our data. This means that with adolescent samples, it does not seem appropriate to compute a global score of well-being from the WB-Pro. Nonetheless, future research should verify if this result depends on developmental factors or if it could be the result of the relatively small number of participants and/or unstable specifics. We also tested the fit of the 48-item model that showed similar results (CFI =.924, TLI =.912, RMSEA =.045).

Table S3a. Latent correlations of the 15 factors of the WB-Pro - CFA solution

	Autonomy	Clear Thinking	Competence	Emotional Stability	Empathy	Engagement	Meaning	Optimism	Positive Emotions	Positive Relations	Prosocial Behavior	Resilience	Self-acceptance	Self-esteem	Vitality
Autonomy	1	**.57**	**.61**	**.44**	-.04	**.72**	**.51**	**.60**	**.53**	**.44**	.09	**.54**	**.54**	**.50**	**.52**
Clear Thinking		1	**.74**	**.76**	-.11	**.68**	**.55**	**.61**	**.54**	**.41**	**.14**	**.60**	**.60**	**.63**	**.52**
Competence			1	**.61**	-.05	**.84**	**.60**	**.72**	**.56**	**.32**	**.18**	**.63**	**.58**	**.72**	**.59**
Emotional Stability				1	**-.20**	**.53**	**.46**	**.58**	**.52**	**.29**	.04	**.70**	**.61**	**.48**	**.52**
Empathy					1	.08	.10	.04	.02	**.20**	**.67**	**-.27**	**-.22**	.00	.01
Engagement						1	**.67**	**.73**	**.75**	**.46**	**.29**	**.59**	**.63**	**.67**	**.73**
Meaning							1	**.87**	**.64**	**.38**	**.21**	**.52**	**.52**	**.61**	**.61**
Optimism								1	**.73**	**.39**	**.15**	**.63**	**.54**	**.67**	**.66**
Positive Emotions									1	**.52**	**.18**	**.63**	**.58**	**.66**	**.80**
Positive Relations										1	**.28**	**.27**	**.32**	**.40**	**.38**
Prosocial Behavior											1	-.06	-.02	**.17**	**.18**
Resilience												1	**.57**	**.53**	**.62**
Self-acceptance													1	**.63**	**.52**
Self-esteem														1	**.56**
Vitality															1

Note. In bold significant values for $p < .05$

Table S3b. Latent correlations of the 15 factors of the WB-Pro - ESEM solution

	Autonomy	Clear Thinking	Competence	Emotional Stability	Empathy	Engagement	Meaning	Optimism	Positive Emotions	Positive Relations	Prosocial Behavior	Resilience	Self-acceptance	Self-esteem	Vitality
Autonomy	1	**.45**	**.42**	**.36**	-.14	**.41**	**.39**	**.47**	**.35**	**.39**	**.11**	**.40**	**.49**	**.39**	**.41**
Clear Thinking		1	**.38**	**.39**	**-.23**	.25	**.38**	**.47**	**.31**	**.36**	**.17**	**.41**	**.54**	**.54**	**.38**
Competence			1	**.30**	.00	.14	**.37**	**.36**	**.39**	.10	.09	**.25**	**.40**	**.35**	**.35**
Emotional Stability				1	**-.20**	**.34**	**.29**	**.40**	**.24**	.12	**.04**	**.49**	**.37**	**.26**	**.29**
Empathy					1	.07	.01	-.04	-.02	.11	**.48**	**-.29**	**-.28**	-.12	-.07
Engagement						1	**.32**	**.51**	.22	**.31**	**.15**	**.42**	.25	**.32**	**.41**
Meaning							1	**.64**	**.45**	**.29**	**.19**	**.32**	**.39**	**.43**	**.45**
Optimism								1	**.44**	**.38**	.08	**.51**	**.45**	**.52**	**.51**
Positive Emotions									1	**.33**	**.13**	**.35**	**.47**	**.45**	**.59**
Positive Relations										1	**.27**	**.24**	**.32**	**.34**	**.35**
Prosocial Behavior											1	-.07	.03	**.19**	**.15**
Resilience												1	**.40**	**.36**	**.47**
Self-acceptance													1	**.54**	**.44**
Self-esteem														1	**.39**
Vitality															**1**

Note. In bold significant values for $p < .05$

Table S4a. Dimension reduction analysis, eigenvalues, and canonical correlations

Root No.	Canon Cor.	Sq. Cor	Eigenvalue	Wilks' L.	F	G.L. num.	G.L. den.	Sign.
**1**	**.858**	**.735**	**2.780**	**.070**	**5.472**	**18.000**	**3033.648**	**.000**
2	.597	.356	.553	.266	2.990	154.000	2809.591	.000
3	.494	.244	.323	.414	2.318	13.000	2584.000	.000
4	.405	.164	.196	.547	1.884	108.000	2356.716	.000
5	.374	.140	.163	.655	1.614	88.000	2127.512	.000
6	.313	.098	.109	.761	1.297	7.000	1896.045	.052
7	.265	.070	.076	.844	1.040	54.000	1661.775	.396
8	.226	.051	.054	.908	.796	4.000	1423.796	.815
9	.142	.020	.021	.957	.517	28.000	118.437	.983
10	.124	.015	.016	.977	.431	18.000	928.209	.981
11	.076	.006	.006	.992	.266	1.000	658.000	.988
12	.047	.002	.002	.998	.185	4.000	33.000	.946

Note. S4a, S4b and S4c refer to Canonical correlation analysis. Analysis conducted with measured variables in SPSS. First set of variables: correlates of well-being. Second set of variables: 15 dimensions of the WB-Pro. In bold the retained root

Table S4b. Proportion of shared variance

Root No.	Shared variance of set 1 with its own variate	Redundancy for set 1 (Shared variance of set 1 with its opposite variate)	Shared variance of set 2 with its own variate	Redundancy of set 2 (Shared variance of set 2 with its opposite variate)
**1**	**.309**	**.227**	**.405**	**.298**
2	.073	.026	.077	.027
3	.064	.016	.041	.010
4	.064	.011	.055	.009
5	.118	.017	.065	.009
6	.073	.007	.052	.005
7	.055	.004	.030	.002
8	.051	.003	.042	.002
9	.040	.001	.029	.001
10	.065	.001	.049	.001
11	.038	.000	.036	.000
12	.048	.000	.030	.000

Note. Tables S4a, S4b and S4c refer to Canonical correlation analysis. Analysis conducted with measured variables in SPSS. First set of variables: correlates of well-being. Second set of variables: 15 dimensions of the WB-Pro. In bold the retained root

Table S4c. Canonical loadings and cross-loadings for root number 1

Variable name	Canonical loadings	Canonical cross-loadings
Variable Set 1: Well-being correlates		
SWLS	.86	.74
Satisfaction with perceived support	.70	.60
Satisfaction with family relations	.56	.48
Basic Needs-Competence	.54	.46
Basic Needs-Relations	.53	.45
Regulatory self-efficacy	.52	.45
Satisfaction with acknowledgment received	.52	.44
Satisfaction wth actual situation	.50	.43
Satisfaction with decisional autonomy	.50	.43
Satisfaction with school peers relations	.48	.41
School satisfaction	.42	.36
Basic Needs-Autonomy	.40	.34
Variable Set 2: WB-Pro dimensions		
Positive emotions	.82	.70
Engagement	.75	.65
Optimism	.75	.64
Meaning	.72	.62
Positive relations	.71	.61
Clear thinking	.71	.60
Competence	.70	.60
Self-esteem	.68	.58
Vitality	.65	.56
Self-acceptance	.63	.54
Autonomy	.58	.50
Resilience	.55	.47
Emotional stability	.53	.46
Prosocial behavior	.22	.19
Empathy	.02	.02

Note. Tables S4a, S4b and S4c refer to Canonical correlation analysis. Analysis conducted with measured variables in SPSS. First set of variables: correlates of well-being. Second set of variables: 15 dimensions of the WB-Pro. In bold the retained root

Table S5. Activities of the "Take care of yourself"-program and their link to activities in previous programs to enhance well-being

**Activities from previous scientific literature that inspired the program "Take care yourself"**	**Activities proposed in the program "Take care yourself"**
List at least 20 activities that we enjoy doing. If we find it difficult to come up with 20, let's think about activities that we enjoyed in the past and that we might still enjoy doing now.<br><br>*http://www.ccm-network.it/documenti_Ccm/prg_area5/2005-manuale-scuola-depressione.pdf*<br><br>*Veltro et al., 2015 p. 174*	1) Work in pairs – identify and share at least three daily activities that you do with interest and involvement.<br><br>2) Choose at least one activity to pursue with dedication and commitment, and write it down in your notebook
Notice and appreciate the good stuff in life.<br><br>*Burckhardt et al., 2015 p. 4*	Identify and write down in your notebook five things you are grateful for and five good things in your life
Values' is the identification of one's values or<br><br>the personality qualities one wishes to espouse. 'Committed action' is taking action that is consistent with one's values.<br><br>*Burckhardt et al., 2016 p. 44*	1) Define what values are and identify at least five of the most important ones in life.<br><br>2) Address the topic of values, choose a single definition of value, combine the values identified in the two subgroups, and draw up a single list of values.<br><br>3) Rank the values that emerged in class in order of importance. With regard to the first one, indicate which actions in your daily life show that it deserves first place among your values
Developing and optimizing the positive individual characteristics – my five signature strengths.<br><br>*Freire et al., 2018 p. 176*	1) Identify and write down three strengths in your notebook<br><br>2) Ask two people you trust to list three strengths they see in you and write them down in your notebook

1) Each day record something<br><br>to be grateful for and<br><br>consider the reasons to<br><br>be grateful<br><br>2) Think about things/people<br><br>to be grateful for and<br><br>practise expressing gratitude<br><br>by writing a letter.<br><br>*Putwain, Gallard, & Beaumont, 2019 pp. 52-53*	1) Identify and write down in your notebook five things you are grateful for and five good things in your life<br><br>2) From today until our next meeting, practice five acts of kindness. How did they make you feel? Write them down in your notebook
1) We can allow and be compassionate towards difficult psychological experiences, which are evolved or learnt responses intended to protect us, are universal,<br>and inherently harmless<br><br>2) We may observe patterns of the same thoughts, feelings, and behaviors, which<br><br>have developed over time (long-standing patterns can be construed as aspects of personality)<br><br>3) Psychological experiences (including familiar patterns of these) can be biased and urge us to behave in ways that are unhelpful for us in the long-term, but we can avoid becoming fused with them and/or change our response to them.<br><br>*Perkins et al., 2021 p. 951*	1) Describe yourself, portraying the best and most realistic version of yourself that you can imagine right now.<br><br>2) For the next meeting, think of something wonderful that you would like to happen within the year. How does this thought make you feel?" Write it down in your notebook.<br><br>3) Create your own personal motto!" Write it down in your notebook; take a photo of it with your cell phone...open the photo whenever you want to remember who you are and how much you are worth

Table S6. Protocol fidelity

	Type of design	Focus of the intervention	Sessions	Delivery-time	Teachers	Focus of each session	Organization of each session	Methodology	Activities for the activation phase	Order of sessions
Original design	Classes to be assigned at random to intervention and control conditions	6 primary well-being dimensions<br><br>+ 2 secondary well-being dimensions	Six to eight<br><br>1-hour sessions	During class-time	Welcomed as members of the group	Each session was focused on at least one of the primary well-being dimensions (optional: additional focus on a secondary well-being dimension)	Opening (framework for the session)<br><br>Activation (from experience to theoretical constructs)<br><br>Closure (debriefing)<br><br>Prescription (homework to translate the training into practice)	Co-construction of meaning through dialogue and debate<br><br>Individual or couple or small group work<br><br>Circle time discussion<br><br>Slide Presentation	Activities were connected to at least one of the primary well-being dimensions (optional: additional connection to a secondary well-being dimension)	No specific order of sessions was anticipated

Design Implementation	No (schools assigned classes to the conditions on the basis of curricular teachers' availability)	yes	six 1-hour sessions	yes	yes	yes	yes	yes	yes	- Positive emotions + Pro-social behavior<br><br>- Competence + Self-esteem<br><br>- Engagement<br><br>- Meaning<br><br>- Optimism<br><br>- Self-esteem + Self-acceptance
Reason for change in respect of the original design		N/A	Principals required no more than 6 sessions to avoid interference with other school activities	N/A		N/A	N/A		N/A	We used the above order for all classes to avoid introducing differences among the classes

Table S7. Intraclass Correlation Coefficient for Group and Class

Variables at pre-test for Group (Experimental-Control)	ICC1	ICC2
Competence	0.018	0.469
Engagement	0.084	0.809
Positive emotions	0.022	0.511
Meaning	0.017	0.445
Optimism	0.125	0.870
Self-esteem	0.011	0.344
Positive relations	0.111	0.854
Self-acceptance	0.031	0.595
Prosocial behavior	0.116	0.860
**Variables at pre-test for Class (10 Classes)**	**ICC1**	**ICC2**
Competence	0.107	0.529
Engagement	0.087	0.471
Positive emotions	0.173	0.663
Meaning	0.048	0.321
Optimism	0.043	0.297
Self-esteem	0.141	0.604
Positive relations	0.121	0.564
Self-acceptance	0.117	0.552
Prosocial behavior	0.116	0.860

Nota. ICC1= Individual-level variance that can be explained by group membership; ICC2= The reliability of the group means.

Table S8. Intervention fidelity

General dimension	Specific dimension	Barrier	Adaptation	Measure
**Adherence to the intervention**				
	Session duration	Delays in class change	Acceptable timetable fit: 50 minutes	
	Session duration	In the pilot study, sessions occurring during the first or last school hours were disturbed by students entering school late or students authorized to leave school early due to commuting	The first and last school hours were avoided in the intervention study	
	Proposed activities	A few curricular teachers/teaching assistants were not supportive of the program	We stressed that curricular teachers who were not willing to actively participate were not obliged to take part in the sessions	
**Quality**				
	Knowledge and experience of the interventionist on the well-being constructs and activities implemented in the program	none	N/A	High
	Engagement and enthusiasm of the interventionist	none	N/A	High
**Dosage**				
	Sessions	none	N/A	All the 6 sessions were implemented in all the classes involved

	Students' exposure to the program	Due to contingent issues, not all students attended all sessions	N/A	77% of the students participated in at least 4 out 6 sessions
**Students' responsiveness**				
	Satisfaction with activities questionnaire	none	N/A	The students reported satisfaction with the program (e.g., mean = 6.55, d.s. = 2.25);
	Teachers' perception of students' interest and engagement	none	N/A	Teachers reported a high interest and engagement of the students for the program
	Students' participation in the debriefing	Some students were shy or not willing to share with everybody	The psychologist stressed that each one could share or not share freely	
**Intervention Monitoring**				
	Fidelity checklist	none	N/A	After three sessions
	Brief consultation with school delegates	none	N/A	After each session
	Supervision with the research coordinator	none	N/A	After each session

Table S9. Fidelity Checklist

School ______________________	*Class* ______________	*Total students of the class* ____
Number of session's participants _______	*Session date* ___/___/___<br>*Time: Start* ________ *Finish* ________	
Session Number: ______	*Primary Dimension:* __________________________________	

Elements	Score	Notes *(Barriers/Possible solutions)*
	Part 1	
**Adherence to intervention**		
Activities implemented as intended	0=No; 1=Partially; 2=Yes	
Methodology adequate to activities (e.g. individual/pair / small group work)	0=No; 1=Partially; 2=Yes	
Completeness of intervention delivery	0=Not completed; 1=Partially; 2=Completed	
**Dosage**		
Respect of sessions frequency (7-10 days)	0=No; 1=Yes	
Respect of time intervention (≥50 minutes workable)	0=No; 1=Yes	
Duration of intervention (respect to activities)	0=Too short; 1=Adequate; 0=Too long	
Intensity intervention	0=Low; 1=Adequate; 0=High	
Total score __________ ***(aim ≥ 8)***		
	Part 2	
**Students' responsiveness** (1 = very poor; 2 = poor; 3 = acceptable; 4 = good; 5 = excellent)		
Respect of activities between sessions	1 – 2 – 3 – 4 – 5	
Students' engagement	1 – 2 – 3 – 4 – 5	
**Quality**		
Ability to conduct the intervention	1 – 2 – 3 – 4 – 5	
Enthusiasm to conduct the intervention	1 – 2 – 3 – 4 – 5	
Achieving intervention results	1 – 2 – 3 – 4 – 5	
Overall quality	1 – 2 – 3 – 4 – 5	
Total score __________ ***(aim ≥ 24)***		

Table S10. The satisfaction questionnaire means for the schools involved in the intervention study

	Technical school		Pedagogical lyceum			
	M	S.D.	M	S.D.	$F_{(1, 75)}$	p
1) Il percorso svolto ha soddisfatto le tue aspettative? (Tr. Did the program meet your expectations?)	7.00	1.60	5.16	2.69	14.18	.00
2) Complessivamente, ritieni ti sia stato utile prendere parte alle attività proposte? (Tr. Overall, do you think it was useful for you to take part in the proposed activities?)	7.02	1.85	5.25	2.44	13.15	.00
3) Le attività svolte sono state utili per aiutarmi nello sperimentare emozioni positive (Tr. The activities carried out were useful in helping me to experience positive emotions)	6.20	2.34	4.94	2.34	5.44	.02
4) Le attività svolte sono state utili per sentirmi una persona capace nelle cose che faccio (Tr. The activities carried out were useful in making me feel capable in the things I do)	6.13	2.21	4.91	2.54	5.08	.03
5) Le attività svolte sono state utili per avere un atteggiamento positivo nei confronti del futuro (Tr. The activities carried out were useful in fostering a positive attitude towards the future)	6.31	2.18	5.12	2.55	4.80	.03
6) Le attività svolte sono state utili per avere la sensazione che ci sia valore in ciò che faccio nella vita (Tr. The activities carried out were useful in giving me the feeling that there is value in what I do in life)	6.24	2.25	5.09	2.52	4.43	.04
7) Le attività svolte sono state utili per valutare me stessa/o positivamente (Tr. The activities carried out were useful for evaluating myself positively)	6.27	2.09	5.56	2.51	1.79	.19

8) Le attività svolte sono state utili per sentirmi coinvolta/o nella maggior parte delle attività che svolgo (Tr. The activities carried out were useful in making me feel involved in most of the activities I do)	6.22	2.36	4.94	2.55	5.17	.03
9) Le attività svolte sono state utili per migliorare il mio benessere (Tr. The activities carried out were useful for improving my wellbeing)	6.22	2.12	5.06	2.55	4.72	.03
15) Quanto ritieni adeguato il supporto ricevuto dalla conduttrice? (ad es. ha risposto alle tue domande, ha chiarito i tuoi dubbi, ha facilitato la tua partecipazione)? (Tr. How adequate do you consider the support received from the facilitator? (e.g. did she answer your questions, clarify your doubts, facilitate your participation)?)	7.33	1.54	5.50	2.69	14.37	.00
17) Alla fine di queste attività, quanto ti ritieni complessivamente soddisfatta/o? (Tr. At the end of these activities, how satisfied are you overall?)	7.09	2.00	5.06	2.31	16.86	.00
18) Quanto consiglieresti questo percorso ad altri studenti e studentesse della tua scuola? (Tr. How much would you recommend this program to other students at your school?)	7.47	2.07	5.16	2.84	17.04	.00

Table S11. Means and standard deviations for intervention and control groups at pre- and post-test

Dimensions	Group	Time	Count	Mean	S.D.
Meaning[1]	Control	Post	45	6.22	1.88
		Pre	45	6.53	1.89
	Intervention	Post	49	6.16	1.49
		Pre	49	6.03	1.66
	Total	Post	94	6.19	1.68
		Pre	94	6.27	1.78
Competence[1]	Control	Post	45	6.60	1.38
		Pre	45	6.47	1.15
	Intervention	Post	49	6.52	1.40
		Pre	49	6.13	1.28
	Total	Post	94	6.56	1.38
		Pre	94	6.29	1.22
Self-esteem[1]	Control	Post	44	6.88	1.39
		Pre	44	6.69	1.33
	Intervention	Post	49	6.25	1.59
		Pre	49	6.35	1.29
	Total	Post	93	6.55	1.52
		Pre	93	6.51	1.31
Optimism[1] **	Control	Post	45	5.90	1.84
		Pre **	45	6.50	1.55
	Intervention	Post	49	5.65	1.61
		Pre **	49	5.63	1.49
	Total	Post	94	5.77	1.72
		Pre	94	6.04	1.57
Engagement[1] *	Control	Post	44	6.50	1.40
		Pre *	44	6.65	1.58
	Intervention	Post	48	6.20	1.23
		Pre *	48	5.90	1.55
	Total	Post	92	6.34	1.31
		Pre	92	6.26	1.60
Positive Emotions[1]	Control	Post	45	6.29	1.67
		Pre	45	5.78	1.53
	Intervention	Post	49	5.61	1.78
		Pre	49	5.34	1.43
	Total	Post	94	5.94	1.75
		Pre	94	5.55	1.49

Prosocial behavior[2] **	Control	Post	44	7.14	1.45
		Pre **	44	6.30	1.65
	Intervention	Post	48	6.64	1.49
		Pre **	48	5.40	1.57
	Total	Post	92	6.88	1.48
		Pre	92	5.83	1.66
Self-acceptance[2]	Control	Post	43	5.72	1.68
		Pre	43	6.46	1.54
	Intervention	Post	48	6.06	1.47
		Pre	48	6.09	1.35
	Total	Post	91	5.90	1.58
		Pre	91	6.26	1.45
Positive relations[2] **	Control	Post	45	7.11	1.46
		Pre **	45	6.86	1.25
	Intervention	Post	47	6.29	1.53
		Pre **	47	6.16	1.35
	Total	Post	92	6.69	1.54
		Pre	92	6.50	1.34

Note. [1] Primary dimension of the training to enhance well-being; [2] Secondary dimensions of the training to enhance well-being relevant to the adolescence phase; * Statistically different means between Control and Intervention groups at the pre-test measure for $p<.05$; ** Statistically different means between Control and Intervention groups at the pre-test measure for $p<.01$;

Table S12. Estimated marginal means for intervention and control groups

Dependent variable	Group	Mean	SE	df	95% CI	
					Lower	Upper
Meaning[1] post	Control	6.11	.20	90	5.71	6.52
	Intervention	6.33	.20	90	5.95	6.72
Competence[1] post	Control	6.52	.22	9.78	6.03	7.01
	Intervention	6.64	.22	8.32	6.14	7.14
Self-esteem post[1]	Control	6.84	.21	89	6.43	7.24
	Intervention	6.36	.20	89	5.97	6.75
Optimism[1] post	Control	5.62	.21	90	5.19	6.04
	Intervention	5.96	.20	90	5.56	6.37
Engagement[1] post	Control	6.33	.21	7.35	5.85	6.82
	Intervention	6.34	.20	6.18	5.85	6.83
Positive emotion[1] post	Control	6.22	.28	9.84	5.59	6.86
	Intervention	5.74	.28	8.15	5.10	6.39
Prosocial behavior[2] post	Control	6.98	.22	88	6.54	7.42
	Intervention	6.73	.21	88	6.31	7.15
Self-acceptance[2] post	Control	5.66	.24	10.52	5.13	6.19
	Intervention	6.20	.23	8.30	5.67	6.73
Positive relations[2] post	Control	6.90	.22	5.96	6.37	7.44
	Intervention	6.48	.22	4.70	5.91	7.05

Note. Measures at pre-test as covariates. [1]. Primary dimension of the training to enhance well-being; [2]. Secondary dimensions of the training to enhance well-being relevant to the adolescent phase

Table S13. Levene test for equality of variances as assumption for group mean comparisons (independent samples t-test in the pilot study)

**Pre-test (T1)**	**F**	**d.f.1**	**d.f.2**	**p**
Autonomy	0.03	1	23	0.86
Emotional stability	2.06	1	23	0.17
Positive emotions	2.31	1	23	0.14
Positive relations	2.18	1	23	0.15
Resilience	0.34	1	23	0.57
Self-acceptance	0.08	1	22	0.77
Self-esteem	1.62	1	23	0.22
Vitality	0.94	1	23	0.34
Competence	2.80	1	22	0.11
Clear thinking	0.31	1	23	0.58
Meaning	2.69	1	23	0.12
Optimism	1.30	1	23	0.27
Prosocial Behavior	0.99	1	23	0.33
Empathy	0.28	1	23	0.60
Engagement	1.09	1	23	0.31
**Post-test (T2)**				
Autonomy	0.17	1	23	0.68
Emotional stability	0.28	1	23	0.60
Positive emotions	0.03	1	23	0.86
Positive relations	0.09	1	23	0.77
Resilience	2.23	1	23	0.15
Self-acceptance	0.61	1	23	0.44
Self-esteem	1.01	1	23	0.32
Vitality	0.01	1	23	0.93
Competence	0.82	1	22	0.37
Clear thinking	0.62	1	23	0.44
Meaning	0.52	1	23	0.48
Optimism	1.11	1	23	0.30
Prosocial Behavior	0.72	1	23	0.41
Empathy	4.49	1	23	0.05
Engagement	0.04	1	23	0.84

Table S14. Tests of group equivalence before the training (pre-test) and mean differences post-training (post-test) in the pilot sample

Pre-test (T1)	Group	N	Mean	s.d.	t-test	d.f.
Autonomy	Experimental	14	6.71	1.57	-.70	23
	Control	11	7.15	1.54		
Emotional stability	Experimental	14	5.67	1.96	-1.06	23
	Control	11	6.36	1.08		
Positive emotions[1]	Experimental	14	6.71	.94	-.76	23
	Control	11	7.06	1.35		
Positive relations[2]	Experimental	14	7.43	1.18	1.37	23
	Control	11	6.82	1.01		
Resilience	Experimental	14	5.55	1.34	-2.27*	23
	Control	11	6.85	1.52		
Self-acceptance[2]	Experimental	14	6.16	1.28	-.77	22
	Control	10	6.58	1.31		
Self-esteem[1]	Experimental	14	6.90	1.29	.09	23
	Control	11	6.85	1.70		
Vitality	Experimental	14	5.12	2.19	-1.60	23
	Control	11	6.42	1.77		
Competence[1]	Experimental	13	7.13	1.33	.89	22
	Control	11	7.55	.86		
Clear thinking	Experimental	14	6.36	1.24	2.10*	23
	Control	11	7.43	1.32		
Meaning[1]	Experimental	14	6.48	1.85	1.56	23
	Control	11	7.49	1.21		
Optimism[1]	Experimental	14	6.48	1.30	.43	23
	Control	11	6.73	1.60		
Prosocial Behavior[2]	Experimental	14	6.69	1.43	-.80	23
	Control	11	7.12	1.21		
Empathy	Experimental	14	5.52	1.51	-.37	23
	Control	11	5.30	1.44		
Engagement	Experimental	14	6.67	1.53	1.42	23
	Control	11	7.45	1.15		
**Post-test (T2)**	**Group**	**N**	**Mean**	**s.d.**	**t-test**	**d.f.**
Autonomy	Experimental	14	6.69	1.47	-1.75	23
	Control	11	7.64	1.15		
Emotional stability	Experimental	14	5.71	1.83	-1.44	23
	Control	11	6.70	1.50		
Positive emotions[1]	Experimental	14	6.43	1.79	-1.41	23
	Control	11	7.36	1.43		

Positive relations[2]	Experimental	14	7.25	1.18	.00	23
	Control	11	7.25	1.01		
Resilience	Experimental	14	6.14	2.00	-1.11	23
	Control	11	6.91	1.23		
Self-acceptance[2]	Experimental	14	6.68	1.34	-.48	23
	Control	11	6.95	1.54		
Self-esteem[1]	Experimental	14	6.74	1.91	-.94	23
	Control	11	7.33	.98		
Vitality	Experimental	14	5.79	1.61	-1.86	23
	Control	11	6.94	1.45		
Competence[1]	Experimental	14	6.74	1.73	1.22	22
	Control	10	7.47	.89		
Clear thinking	Experimental	14	6.52	1.70	1.69	23
	Control	11	7.53	1.10		
Meaning[1]	Experimental	13	6.57	1.92	1.24	23
	Control	11	7.42	1.36		
Optimism[1]	Experimental	14	6.19	1.99	1.23	22
	Control	11	7.03	1.22		
Prosocial Behavior[2]	Experimental	14	6.69	1.43	-.80	23
	Control	11	7.12	1.21		
Empathy	Experimental	14	5.80	1.09	-.82	23
	Control	11	5.36	1.58		
Engagement	Experimental	14	6.31	1.49	1.25	23
	Control	11	7.03	1.35		

Note. * $p<.05$; [1] Primary dimension of the training to enhance well-being; [2] Secondary dimensions of the training to enhance well-being relevant to the adolescence phase

Table S15. Paired t-test for Control and Experimental groups for the pilot sample

Group	Well-being dimension	Time	Mean	N	s.d.	t-test	d.f.
Control	Autonomy	pre-test	7.15	11	1.54	-1.57	10
		post-test	7.64	11	1.15		
	Emotional stability	pre-test	6.36	11	1.8	-.89	10
		post-test	6.70	11	1.50		
	Positive emotions[1]	pre-test	7.6	11	1.35	-1.39	10
		post-test	7.36	11	1.43		
	Positive relations[2]	pre-test	6.82	11	1.1	-1.51	10
		post-test	7.25	11	1.1		
	Resilience	pre-test	6.85	11	1.52	-.17	10
		post-test	6.91	11	1.23		
	Self-acceptance[2]	pre-test	6.58	10	1.31	-1.99	9
		post-test	6.98	10	1.63		
	Self-esteem[1]	pre-test	6.85	11	1.70	-1.81	10
		post-test	7.33	11	.98		
	Vitality	pre-test	6.42	11	1.77	-1.96	10
		post-test	6.94	11	1.45		
	Competence[1]	pre-test	7.55	10	.86	-.19	9
		post-test	7.47	10	.89		
	Clear Thinking	pre-test	7.43	11	1.31	-.44	10
		post-test	7.52	11	1.10		
	Meaning[1]	pre-test	7.48	10	1.21	.29	10
		post-test	7.42	10	1.36		
	Optimism[1]	pre-test	6.63	11	1.60	-1.49	10
		post-test	7.03	11	1.22		
	Prosocial Behavior[2]	pre-test	7.24	11	.99	.36	10
		post-test	7.12	11	1.21		
	Empathy	pre-test	5.30	11	1.39	-.22	10
		post-test	5.36	11	1.29		
	Engagement	Pre-test	7.45	11	1.15	1.55	10
		pre-test	7.03	11	1.35		
Experimental	Autonomy	pre-test	6.71	14	1.57	.06	13
		post-test	6.69	14	1.47		
	Emotional stability	pre-test	5.67	14	1.96	-.18	13
		post-test	5.71	14	1.83		
	Positive emotions[1]	pre-test	6.71	14	.94	.59	13
		post-test	6.43	14	1.79		
	Positive relations[2]	pre-test	7.43	14	1.18	.69	13
		post-test	7.25	14	1.18		

Resilience	pre-test	5.55	14	1.34	-.98	13
	post-test	6.14	14	2.00		
Self-acceptance[2]	pre-test	6.16	14	1.28	-1.43	13
	post-test	6.68	14	1.34		
Self-esteem[1]	pre-test	6.90	14	1.29	.52	13
	post-test	6.74	14	1.91		
Vitality	pre-test	5.12	14	2.19	-1.36	13
	post-test	5.79	14	1.61		
Competence[1]	pre-test	7.13	13	1.33	1.02	12
	post-test	6.74	13	1.63		
Clear Thinking	pre-test	6.36	14	1.24	-.54	13
	post-test	6.52	14	1.70		
Meaning[1]	pre-test	6.48	14	1.85	-.24	13
	post-test	6.57	14	1.92		
Optimism[1]	pre-test	6.48	14	1.30	1.15	13
	post-test	6.19	14	1.99		
Prosocial Behavior[2]	pre-test	6.83	14	1.53	.50	13
	post-test	6.69	14	1.43		
Empathy	pre-test	5.52	14	1.53	-1.02	13
	post-test	5.80	14	1.49		
Engagement	Pre-test	6.67	14	1.53	.87	13
	post-test	6.30	14	1.48		

Note. [1] Primary dimension of the training to enhance well-being; [2] Secondary dimensions of the training to enhance well-being relevant to the adolescence phase

Supplementary Figures

Figure S1. Histogram graphs

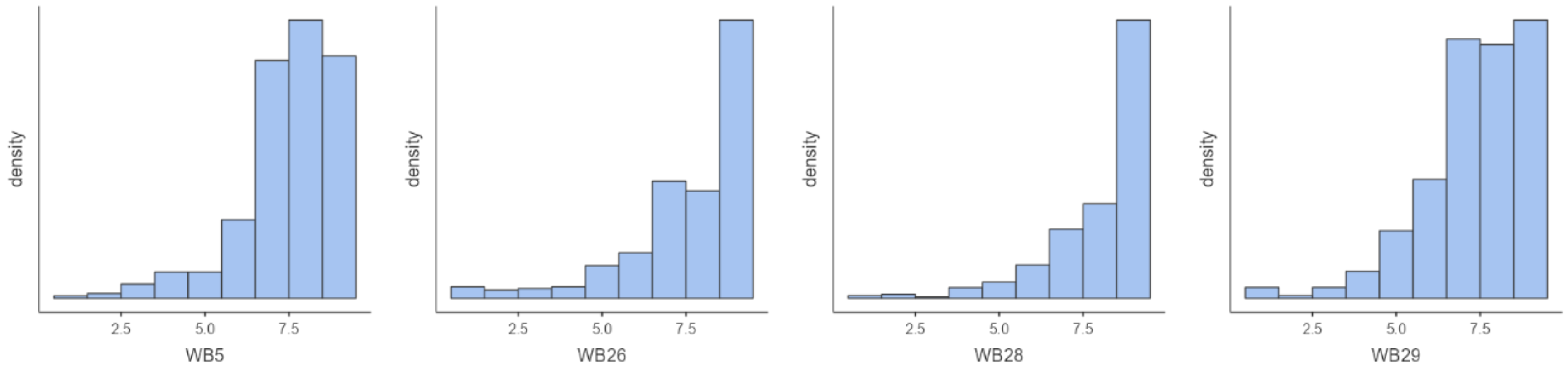

The kurtosis and skewness values of almost all the items are within the –2 and +2 range of normal univariate distribution (George & Mallery, 2010; Gravetter & Wallnau, 2014; Hair, Hult, Ringle & Sarstedt, 2022). Only items WB5, WB26, WB28 and WB29 showed higher values of kurtosis (from 2.20 to 4.36) due to the extreme concentration of scores at the top, leading to a possible ceiling effect. According to Kim (2013), when the sample size is n>300, the histogram graph should be interpreted and an absolute kurtosis values larger than 7 may be used as reference values for determining substantial non-normality. Although values could be considered acceptable, the histograms of items WB26 (It. "Ho delle relazioni strette e su cui posso contare", En. "I have close and secure relationships") and WB28 (It. "Ci sono persone nella mia vita che tengono davvero a me", En. "There are people in my life who really care about me"), both included on Positive relationships scale, do not show a symmetrical bell shape, reducing the ability of this scale to discriminate between individuals.

Figure S2. Plan of the studies

Pilot study (September-November 2024)

Pre-test	Intervention	Post-test	Interviews with teachers
WB-Pro	Six sessions of 60 minutes	WB-Pro<br>SWLS	Changes in the program for the intervention

Intervention study (January-May 2025)

Pre-test	Intervention	Post-test	Interviews with teachers	Follow-up
WB-Pro<br>SWLS	Six sessions of 60 minutes	WB-Pro<br>SWLS<br>Satisfaction questionnaire		WB-Pro Short version<br>SWLS

Figure S3. Flowchart of participants for the pilot study

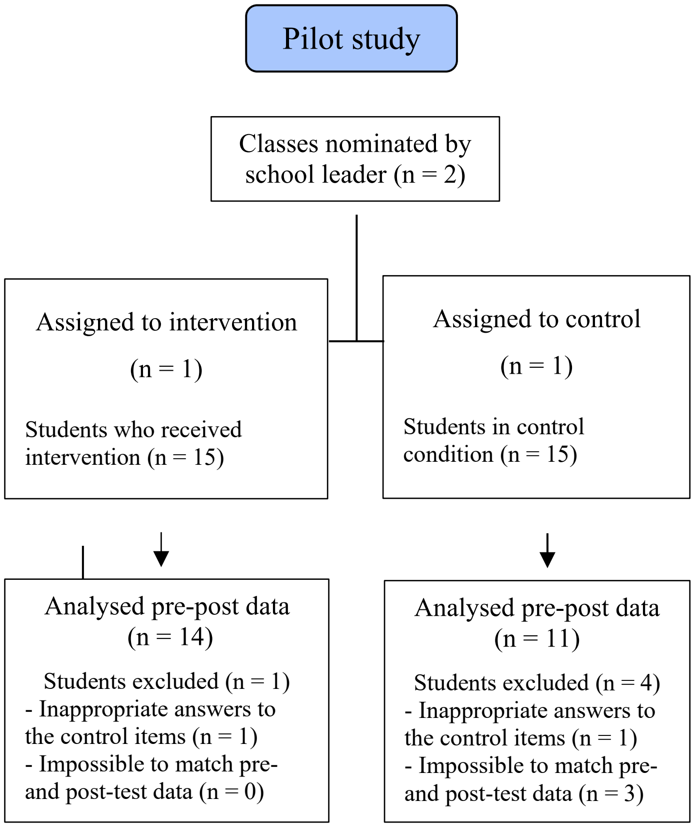

References

Field, A. (2013). *Discovering statistics using SPSS.* Sage Publications.

Gravetter, F., & Wallnau, L. (2014). Essentials of statistics for the behavioral sciences. Wadsworth.

Hair, J.F., Hult, G.T.M., Ringle, C.M., & Sarstedt, M. (2022). *A Primer on Partial Least Squares Structural Equation Modeling (PLS-SEM)*, 3rd ed. Thousand Oaks, CA: Sage.

Kim, H.Y. (2013). Statistical notes for clinical researchers: assessing normal distribution (2) using skewness and kurtosis. *Restor Dentistry Endodontics*, 38(1), 52-54. https://doi.org/10.5395/rde.2013.38.1.52
